# Low-Level Ionizing Radiation Induces Selective Killing of HIV-1-Infected Cells with Reversal of Cytokine Induction Using mTOR Inhibitors

**DOI:** 10.3390/v12080885

**Published:** 2020-08-13

**Authors:** Daniel O. Pinto, Catherine DeMarino, Thy T. Vo, Maria Cowen, Yuriy Kim, Michelle L. Pleet, Robert A. Barclay, Nicole Noren Hooten, Michele K. Evans, Alonso Heredia, Elena V. Batrakova, Sergey Iordanskiy, Fatah Kashanchi

**Affiliations:** 1 Laboratory of Molecular Virology, School of Systems Biology, George Mason University, Manassas, VA 20110, USA; dpinto1@gmu.edu (D.O.P.); cdemarin@gmu.edu (C.D.); tthyvo@gmail.com (T.T.V.); mcowen4@gmu.edu (M.C.); ykim78@gmu.edu (Y.K.); mpleet@gmu.edu (M.L.P.); rbarclay@gmu.edu (R.A.B.); 2 Laboratory of Epidemiology and Population Science, National Institute on Aging, National Institutes of Health, Baltimore, MD 21224, USA; norenhootenn@mail.nih.gov (N.N.H.); me42v@nih.gov (M.K.E.); 3 Institute of Human Virology, University of Maryland School of Medicine, University of Maryland, Baltimore, MD 21201, USA; aheredia@ihv.umaryland.edu; 4 Department of Medicine, University of North Carolina HIV Cure Center; University of North Carolina at Chapel Hill School of Medicine, Chapel Hill, NC 27599, USA; batrakova@unc.edu; 5 Department of Pharmacology and Molecular Therapeutics, Uniformed Services University of the Health Sciences, Bethesda, MD 20814, USA; sergey.iordanskiy@usuhs.edu

**Keywords:** HIV-1, autophagy, extracellular vesicles, latency reversal, Ionizing radiation, cell death, shock and kill, mTOR inhibition, HIV-1 therapy, inflammation

## Abstract

HIV-1 infects 39.5 million people worldwide, and cART is effective in preventing viral spread by reducing HIV-1 plasma viral loads to undetectable levels. However, viral reservoirs persist by mechanisms, including the inhibition of autophagy by HIV-1 proteins (i.e., Nef and Tat). HIV-1 reservoirs can be targeted by the “shock and kill” strategy, which utilizes latency-reversing agents (LRAs) to activate latent proviruses and immunotarget the virus-producing cells. Yet, limitations include reduced LRA permeability across anatomical barriers and immune hyper-activation. Ionizing radiation (IR) induces effective viral activation across anatomical barriers. Like other LRAs, IR may cause inflammation and modulate the secretion of extracellular vesicles (EVs). We and others have shown that cells may secrete cytokines and viral proteins in EVs and, therefore, LRAs may contribute to inflammatory EVs. In the present study, we mitigated the effects of IR-induced inflammatory EVs (i.e., TNF-α), through the use of mTOR inhibitors (mTORi; Rapamycin and INK128). Further, mTORi were found to enhance the selective killing of HIV-1-infected myeloid and T-cell reservoirs at the exclusion of uninfected cells, potentially via inhibition of viral transcription/translation and induction of autophagy. Collectively, the proposed regimen using cART, IR, and mTORi presents a novel approach allowing for the targeting of viral reservoirs, prevention of immune hyper-activation, and selectively killing latently infected HIV-1 cells.

## 1. Introduction 

Human Immunodeficiency Virus Type-1 (HIV-1) continues to be a significant health concern, with approximately 39.5 million worldwide living with the infection [1]. Although combination antiretroviral therapy (cART) yields low viral titers and decreased mortality in HIV-1-infected patients, chronic HIV-1 infection has been demonstrated to produce viral products in anatomical reservoirs (e.g., peripheral blood, organs, and brain) [2,3,4]. These viral products, such as small non-coding RNA, genomic RNA, viral proteins, and virions in patients with chronic HIV-1 (or latent infection), may be directly associated with the development of central nervous system (CNS) impairments known as HIV-1-associated neurocognitive disorders (HAND) in 30% to 60% of infected patients [5,6,7,8,9]. This is evident as the current antiviral regimens are ineffective at preventing and treating HIV-1-related downstream disorders, such as persistent immune activation [10,11], increased mortality [12,13], drug resistance [14,15], and development of HAND [16,17,18,19]. Further, we and others have demonstrated that, despite cART, the HIV-1 transcription machinery is not entirely suppressed during latent infection resulting in low-level viral transcription, even when viral plasma levels are undetectable (<50 copies RNA/mL) [2,20,21,22,23,24,25]. For these reasons, a more broadly encompassing therapeutic strategy is needed to fight latency. 

The inability to eliminate the latent HIV-1 reservoirs from an infected host has led the field to develop a strategy known as “shock and kill,” which utilizes latency-reversing agents (LRAs) to reactivate the virus (i.e., shock) and implements cART (i.e., kill) in combination with immune system priming to eliminate the newly activated infected cells while preventing the spread of infection. To date, LRAs present limited success primarily due to the inefficacy in reducing the size of the latent reservoir [26,27,28,29]. More specifically, it was shown that LRAs are only partially successful at reactivating latent HIV-1, with reactivation occurring only in 5% of cells within the latent reservoir [30]. Therefore, the remaining subpopulations of latently infected T-cells may lay dormant indefinitely, despite the presence of LRAs [30]. Furthermore, LRAs have been associated with increased inflammation and the generation of reactive oxygen species (ROS) [31,32,33]. 

The most commonly used classes of LRAs are histone deacetylase inhibitors (HDACi; panobinostat, romidepsin, and vorinostat), which function by promoting transcriptionally active chromatin, and protein kinase C inhibitors (PKCi; bryostatin-1 and PKC412) which activate the nuclear factor kappa B (NF-kB) pathway [4,34,35,36,37,38,39,40,41]. Additionally, histone methyltransferase inhibitors (HMTi; chaetocin and BIX-01294) and DNA methylation inhibitors (i.e., 5-Azacitidine) are also being investigated as potential LRAs, particularly when used in conjunction with HDACi or PKCi [40]. More recently, activation of Toll-like receptors (TLRs) has been explored as a potential LRA due to reported in vitro and ex vivo effects on HIV-1 reactivation and potentiation of an immune response against HIV-1 [42]. 

One of the major issues in the reactivation of HIV-1 is that the extent of the viral reservoir within the human body is still not fully understood [30,43]. Additionally, drug-based LRAs face issues related to poor delivery to all viral reservoirs. Ionizing radiation (IR) has been proposed to overcome this obstacle as it has the potential to cross anatomical barriers to enter previously inaccessible latent reservoirs, thereby facilitating effective delivery. We have previously demonstrated that the lymphoma therapy-related IR doses can increase basal and activated transcription in latently infected primary T-cells and a humanized mouse model, as measured by an increase in unspliced HIV-1 RNA post-treatment (5–50-fold and 7–100-fold, respectively) [4]. Furthermore, we have shown that IR can selectively induce apoptosis in infected primary T-cells [4]. Another research group has shown IR-mediated viral activation resulting in induced transient low-level viremia in plasma of cART-treated Simian/Human Immunodeficiency Virus (SHIV)-infected macaques [44]. We have also shown that IR can induce transcription of a related retrovirus, the Human T-cell Lymphotropic Virus Type-1, and not that of cellular transcripts, showing the potential selectivity towards viral transcription [45]. Additionally, IR has clinical applications since radiotherapy is considered feasible in patients with HIV-1 and cancer [46]. Altogether, IR has the potential to be used as an LRA with robust reactivating capabilities in infected myeloid and T-cells [4,47]. However, as with other LRAs, there is still potential to induce the production of ROS and cytokines [31,32,33]. This highlights the need for a combinatorial drug regimen with the inclusion of a drug that mitigates the inflammatory potential of current “shock and kill” strategies.

The host cell type may be an important factor in the effectiveness of “shock and kill” approaches to HIV-1 infection. For example, macrophages, which have a lifespan of months to years and display very low turnover rates, even in the presence of oxidative stress, may be more prone to latency [48,49,50]. In addition, macrophages are very resistant to the cytopathic effects of HIV-1 [51]. Recent studies in SIV-infected macaques [52] and in HIV-infected humanized mice [53,54] demonstrate that tissue macrophages are indeed productively infected, and represent a source of rebound viremia upon cessation of cART. Despite limited success with myeloid lineage cells, the induction of latent HIV-1 in infected T-cells is effectively accomplished by broad T-cell activation (i.e., CD3/CD28). Nevertheless, in vivo, this treatment is also accompanied by the rapid increase in pro-inflammatory cytokines (i.e., TNF-α) and potentially severe clinical side effects [55,56,57]. For this reason, it is crucial to incorporate methods to mitigate cytokine release during “shock and kill” therapies. Previous studies have shown that the pro-inflammatory cytokine release (i.e., TNF-α, IFN-γ, and IL-2) associated with LRAs can be controlled by the use of inhibitors against the mammalian target of Rapamycin (mTOR) complexes mTORC1 and mTORC2 [48,58]. In addition to the reported reduction in cytokine release by inhibition of mTORC1, mTORC2 inhibition has been shown to reduce viremia and block HIV-1 entry via CCR5 [48,49,59]. Therefore, therapeutic targeting of mTOR presents multiple potential benefits to latently infected patients as antagonist drugs may be able to control HIV-1 transcriptional activation, restrict translation of viral proteins, and promote clearance of viral products.

The modulation of cellular host processes, such as transcription, translation, and potentially autophagy, by HIV-1, has been of great interest to those investigating the underlying mechanisms supporting long-term latency. Numerous studies have shown that HIV-1 proteins, such as the Trans-Activator of Transcription (Tat), and host proteins, such as AFF4, are involved in the recruitment of transcription factors, such as the super elongation complex, to regulate HIV-1 transcription [25,60,61,62,63,64,65,66,67,68]. Additionally, mTOR has been shown to be involved in the modulation of HIV-1 transcription as inhibition of this target by mTOR inhibitors (mTORi) was reported to suppress HIV-1 replication in humanized mice [59]. Additional research suggests that inhibition of mTOR complexes was associated with decreased phosphorylation of Cyclin-Dependent Kinase 9 (CDK9) in primary CD4 T-cells [69]. Activation of CDK9 is dependent on association with Cyclin T1 to form the positive transcription elongation factor (p-TEFb) [66,70,71,72,73,74], therefore decreased phosphorylation of CDK9 by mTORi effectively lowers HIV-1 transcription.

Efficient translation of HIV-1 mRNA may also be dependent on the activation of mTOR [59,75]. Under normal conditions, mTOR promotes eukaryotic translation initiation factor 4F (eIF4F) complex assembly at the 5’ end of mRNA. In cancer, the mTOR has been shown to be an important player in the dysregulation of cap-dependent translation [76,77]. Use of mTORi has been reported to significantly reduce translation in 20% of cellular proteins in vitro [78], and reduce tumor growth and metastasis by interfering with the phosphorylation of multiple targets, such as eIF4E binding proteins (4E-BP), ribosomal protein S6 kinase B-1 (S6K1), AKT, PKC, and IGF-IR [79,80,81]. Studies have shown that HIV-1 translation initiation may, at least in part, be cap-dependent [75,82,83,84]. Therefore, there is potential in using mTORi drugs as an approach to interfere with viral translation [59]. 

A well-studied function of mTOR is regulation of the autophagy pathway, which is responsible for maintaining cellular homeostasis via regulation of protein degradation and turnover of cellular components. The mTOR complexes can be pharmacologically inhibited by mTORi to promote the autophagy pathway and have been approved for use in the treatment of cancer [79,85,86]. However, HIV-1 proteins (i.e., Tat and Nef) can interact with autophagy by preventing fusion of the autophagosome and lysosome, thereby limiting degradation of associated cargo and hindering cell component recycling [87,88,89,90,91,92,93,94,95,96,97], effects which are downstream of the target of mTORi. The hijacking of the autophagy pathway by HIV-1 may promote viral replication, but also deregulation of cellular homeostasis, as restoring homeostasis through induced autophagy has been demonstrated to be beneficial to the intracellular microenvironment by the increased the clearance of viral proteins, such as Tat [98,99,100,101,102]. The increase in intracellular reactive oxygen species (ROS) and other pro-inflammatory molecules, such as those induced by IR, has been shown to damage cells [47,103,104,105,106]. Cellular stress may be overcome by activation of the autophagy pathway to maintain homeostasis [105,107,108,109,110,111]. Additionally, cells may use peroxisome-related enzymes, such as superoxide dismutase (SOD) and catalase (CAT), to reduce levels of intracellular ROS [112,113,114]. However, during HIV-1 infection, the deregulation of autophagy likely limits the cell’s ability to control inflammatory proteins, suggesting that the use of an autophagy inducer such as mTORi, could be useful in mediating the side effects of IR. 

Extracellular vesicles (EVs) are membrane-bound structures released from numerous cell types, which have been shown to play an important role in cell–cell communication and in mediating viral pathogenesis [24,45,101,115,116,117,118,119]. EVs may be necessary for the transport of signaling molecules, such as cytokines [120], viral proteins [22,116,121], and cellular proteins [45,101,122], which may be found associated to the EV membrane or encapsulated as cargo [120,123]. We have recently shown that IR may have modulatory effects on the biogenesis, packaging of cargo, and secretion of EVs in another retrovirus, known as the Human T-cell Lymphotropic Virus Type-1 (HTLV-1) [45]. These EVs may have potentially inflammatory effects on recipient cells, by carrying the HTLV-1 transactivating protein Tax, an analog protein to HIV-1 Tat [116]. In HIV-1, we have shown that drug treatment, such as cART and type-I interferons, may affect the packaging and secretion of EVs, to carry more viral proteins and RNA [22]. More importantly, we have also shown that these EVs may cause the upregulation of pro-inflammatory cytokines in recipient cells [24]. For these reasons, it was important for us to investigate the EVs generated from HIV-1-infected cells under “shock and kill” treatments and their effects on recipient cells. 

In the present study, we found that various IR doses selectively reduced the survival of infected T-cells and, to a lesser extent, infected myeloid cells. We found that there are inherently different levels of peroxisome-related enzymes (i.e., CAT and SOD) in immune cell types (i.e., myeloid and T-cells), which are altered during HIV-1 infection, potentially contributing to differential susceptibility to IR. To address the potential side effects of IR and accompanying cell death, we have implemented the use of mTORi, specifically Rapamycin (Rapa) and INK128. Our findings suggest that the combination of IR and activation of autophagy (i.e., serum starvation and mTORi) is a strategy that may potentially purge HIV-1 from reservoirs and selectively induce death in HIV-1-infected cells. Additionally, mTORi can lower the release of EV-associated pro-inflammatory cytokine TNF-α, and viral proteins, potentially mitigating the adverse effects of LRA-associated inflammation. These findings provide a foundation for strategies that may be used in future animal models and clinical studies, with the goal of eradicating HIV-1 from an infected individual. 

## 2. Methods

### 2.1. Reagents and Cell Culture Procedures

Uninfected T-cell (CEM), chronically HIV-1-infected T-cell (ACH2), uninfected promonocytic cell (U937), latent HIV-1-infected promonocytic cell (U1), and HIV-1-infected HeLa cells (HLM-1) were cultured in RPMI-1640 medium supplemented with 10% heat-inactivated FBS, 2 mM L-glutamine, 100 U/mL penicillin, and 100 µg/mL streptomycin. Cell starvation was induced by culturing in media, as described above, with 3% FBS for 3 to 5 days at a concentration of 1 × 10^6^ cells/mL. All cells (infected and uninfected) were treated with cART cocktail of protease inhibitor (Indinavir), nucleoside reverse transcriptase inhibitors (Emtricitabine, Tenofovir, and Lamivudine) at a final concentration of 10 µM per drug per treatment (3 days on; 5 days off). Cell lines and antiretrovirals were obtained from the AIDS Reagent Program (National Institutes of Health, Germantown, MD, USA). 

Rapamycin (Rapa) was obtained from MedChem Express (Cat. #: HY-10219; Monmouth Junction, NJ, USA) and INK128 from APExBIO (Cat. #: A8551; Boston, MA, USA). Stocks were reconstituted in 100% dimethyl sulfoxide (DMSO) and diluted to working stock concentrations with 1X phosphate-buffered saline (PBS). Treatments with Rapa or INK128 for cell viability experiments were performed with 1 µL of the drug to deliver the desired concentration. Final DMSO concentrations in drug formulations were at or below 0.01% DMSO. Catalase (Cat) from bovine liver (Cat. #: CAS 9001-05-2) and superoxide dismutase (SOD) from bovine liver (Cat. #: S8160) were obtained from Sigma-Aldrich (St. Louis, MO, USA). Stock enzymatic activity levels for CAT and SOD were at 1200 U/100 µL and 329 U/100 µL, respectively. 

In order to enhance the efficiency of viral protein and cytokine entry into recipient HLM-1 cells, the HLM-1 cells were incubated in a 24-well plate at 40% confluency (1 × 10^5^ cell/well) in 100 µL of supernatant material from PBMC 5, 6, 7, and 8, and with 5 µL of the infection enhancer, Infectin from Virongy LLC (Cat # IF01; Manassas, VA, USA) for a total of 1 hr. After incubation, 900 µl of EV-free media (ultracentrifuged FBS at 100,000× *g* speed for 90 min to remove EVs) was added to each well and allowed to incubate for 72 h. The supernatants of HLM-1 cells were separated from cell pellets. 

### 2.2. Enrichment of EVs and Virions Using Nanotrap Particles (NTs)

Enrichment of EVs or virions is possible via the use of Nanotrap particles (NTs; Ceres Nanosciences, Inc., Manassas, VA, USA), as described previously [22,24,45,117,124,125]. In brief, cell-free supernatant samples (1mL) were mixed with 30 µL of a mixture of NT80 (Cat. #: CN1030) and NT82 (Cat. #: CN2010) in a 30% slurry in 1x PBS (without Calcium and Magnesium), to enrich for EVs. A mixture of NT80, NT82, and NT86 (Cat. #: CN2030) in a 30% slurry in 1 x PBS (without Calcium and Magnesium) was used to enrich for EVs and HIV-1 virions. Enriched EVs or HIV-1 virions were subsequently used for downstream assays, as described previously [126]. 

### 2.3. Human Cohort Information

A subcohort of eight participants was chosen from the Healthy Aging in Neighborhood of Diversity Across the Life Span (HANDLS) study of the National Institute of Aging Intramural Research Program, National Institutes of Health [127]. The Institute Review Board of the National Institute on Environmental Health Sciences (Bethesda, MD, USA) approved the study, and informed written consent was obtained from all participants. PBMCs were obtained from eight HIV-1 positive participants under antiretroviral treatment, with a status of latent or non-progressor. PBMCs were isolated as previously described [128] and stored at −80 °C until use. Information, such as gender and co-infection status (Hepatitis B and C), for each individual is shown in Table 1.

All PBMCs were cultured in media as described above and with IL-2 and PHA every other day for 7 days. Cells were then treated with 0.5 Gy of IR, 50 nM Rapa or 50 nM INK128, and monitored for 12 days before harvesting for analysis by Western blot, and RT-qPCR. 

### 2.4. Cell Viability Assay

Cell viability was assessed to confirm survival rates of HIV-1-infected and uninfected cells after treatment with IR in combination with Rapa or INK128. A concentration of 5 × 10^4^ cells in 100 uL was plated in triplicate on a 96-well plate. After plating and treatment, cells were incubated for 2, 3, or 5 days (according to the therapeutic regimen), and cell viability assessed with 100 uL of Cell-Titer Glo reagent (Cat. #: G7572; Promega, Madison, WI, USA). A GloMax explorer plate reader (Promega) was used to measure relative luminescence units (RLU) resulting from ATP levels in cultured cells. 

### 2.5. X-ray Irradiation

A RS 2000 X-ray Irradiator (Rad Source, Suwanee, GA) was utilized for IR treatments at 160 kV, 25 mA. A total of 5 and 10 Gy doses were used, at a dose rate of 2.42 Gy/min, for cell lines, and 0.5 Gy (2.42 Gy/min) for human PBMC samples. An incubation period of 3 to 5 days post-IR was used for cell lines and 12 days for PBMC samples. 

### 2.6. Isolation of RNA, Generation of cDNA, and Real-Time Quantitative PCR (RT-qPCR)

Total RNA was isolated using Trizol-chloroform per the manufacturer’s instructions (Invitrogen, Carlsbad, CA, USA) and RNA quantified using a NanoDrop 1000 Spectrophotometer (Thermo Scientific, Waltham, MA, USA). Subsequently, the specific TAR reverse (5′-CAA CAG ACG GGC ACA CAC TAC-3′, Tm = 58 °C) and *env* reverse (5′-TGG GAT AAG GGT CTG AAA CG-3′; Tm = 58 °C) primers and GoScript Reverse Transcription System (Promega) were used to generate cDNA. Next, TAR-Reverse: (5′-CAA CAG ACG GGC ACA CAC TAC-3′, Tm = 58 °C) and TAR-Forward (5′-GGT CTC TCT GGT TAG ACC AGA TCT G-3′, Tm = 60 °C) primers were used for RT-qPCR, as described previously [22]. DNA from HIV-1-infected 8E5 cells was used as the quantitative PCR standard, as described previously [22]. 

### 2.7. SDS Page and Western Blot Analysis 

Cells were pelleted, washed with PBS, and resuspended by gentle mixing with lysis buffer ((50 mM Tris–HCl (pH 7.5), 120 mM NaCl, 5 mM EDTA, 0.5% Nonidet P-40, 50 mM NaF, 0.2 mM Na_3_VO_4_, 1 mM DTT, and 1 protease inhibitor cocktail tablet/50 mL (Roche Applied Science, Penzberg, Germany)) and incubated at 4 °C with vortexing every 5 min for 30 min. The lysate was then separated by centrifugation (10,621× *g* for 10 min at 4 °C) and total protein quantitated using Bradford reagent. Samples were loaded onto a 4–20% Tris-glycine gel (Invitrogen) at a protein concentration of 20 μg of lysate in 20 μL total volume (in Laemmli buffer), run at 100 V, and transferred overnight at 50 mA onto PVDF Immobilon membranes (Millipore). Membrane blocking was performed by a 2 h incubation with 5% DIFCO™ Skim Milk (BD) in PBS with 0.1% Tween-20 (PBS-T) at 4 °C. PBS-T was used to rinse membranes before the addition of primary antibodies. Antibodies against TNF-α (Santa Cruz Biotechnology, Dallas, TX, USA; Cat. #: sc-52746), CD63 (System Biosciences, Palo Alto, CA, USA; Cat. #: EXOAB-CD63A-1), CAT (Bio-Rad; Cat. #: VMA00129), and SOD (Bio-Rad; Cat. #: VPA00070) were purchased from Santa Cruz Biotechnology. HIV-1 p24 antibody was obtained from the NIH AIDS Reagent Program (Cat. #: 6457). Densitometry was analyzed using ImageJ software. Densitometry counts were obtained and normalized by subtracting the background of each membrane and then normalized to Actin for each protein. 

### 2.8. PBMC Infection with Dual-Tropic 89.6 HIV-1 and Induction of Latency 

Healthy PBMCs were purchased from Precision Inc. (Cat. #: 9300-10M). Information, such as gender, age, and ethnicity are listed in Table 2. 

All PBMCs were thawed, washed with PBS and incubated in fresh RPMI media supplemented with FBS and antibiotics as described above, and incubated overnight with IL-2 and PHA every other day for 3 days. Subsequently, cells were infected using dual-tropic HIV-1 isolate 89.6 (MOI: 6). Three days after infection, IL-7 and cART (indinavir, emitricibine, lamivudine, and Tenofovir; 10 µM of each drug) were added every other day for 6 days to promote latency. At day 6, we performed our treatment scheme that consists of IR (0.5 Gy), fresh media with 3% FBS (nutrient starvation), and mTORi (Rapa or INK128; 50 nM) single treatment and allowed to incubate for 5 days. Cells were harvested and lysed for analysis by WB. Each lane was loaded with 12.5 µg of lysate sample.

### 2.9. Statistical analysis

Standard deviations (S.D.) and statistical significance were calculated using Microsoft Excel. The statistical significance of quantitative experiments was determined by the two-tailed Student’s T-test. Values could be considered statistically significant (*p*-value ≤ 0.05), of greater significance (*p*-value ≤ 0.01), or of greatest significance (*p*-value ≤ 0.001). 

## 3. Results 

### 3.1. Catalase and Superoxide Dismutase Mediate Survival of Irradiated HIV-1-Infected Cells 

**Figure 1:**
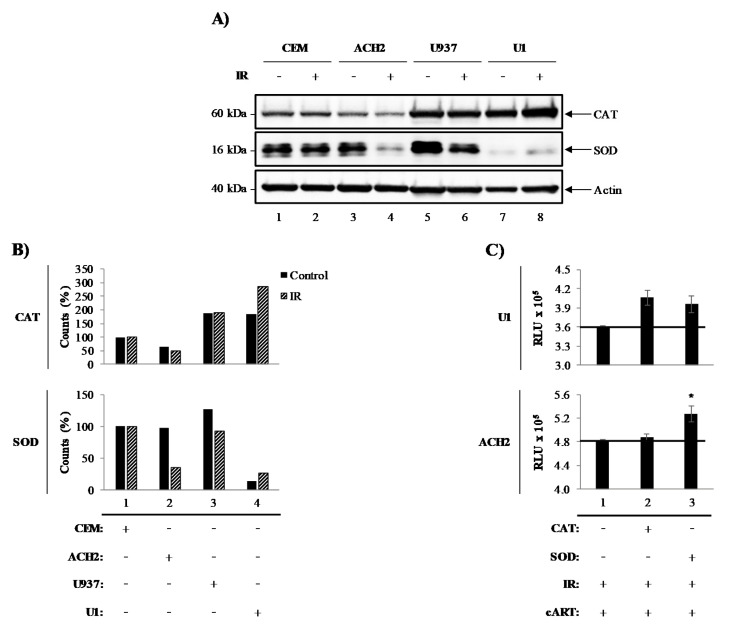
Catalase and superoxide dismutase dictate the survival of irradiated HIV-1-infected cells. (**A**) Chronically HIV-1-infected T-cell line ACH2 and pro-monocytes U1, and corresponding uninfected CEM and U937, treated with 10 Gy of ionizing radiation (IR), lysed (5 days post-IR) and probed for catalase (CAT) and superoxide dismutase (SOD) via Western blot. (**B**) Densitometry analysis of the CAT and SOD bands was performed. (**C**) Two dosages of exogenous CAT (3.08 U) and SOD (16.82 U) were added to U1 and ACH2 cells in two dosages to over the time period of 5 days (Day 1 and 3) to test for cell viability recovery after IR treatment.

We have previously demonstrated that exposure to the therapeutically-related doses of ionizing radiation (IR) induces selective cell death of HIV-1-infected T-cells through modification of tumor suppressor protein (p53) and Poly [ADP-ribose] polymerase 1 (PARP-1). However, characteristically robust resistance to IR induced cell death was reported in HIV-1-infected myeloid cells [4,47]. To investigate the cell-type differences in HIV-1-infected cell survival, we examined the levels of the intracellular pro-survival enzymes, catalase (CAT) and superoxide dismutase (SOD), in HIV-1-infected myeloid (U1) and T-cells (ACH2), and their uninfected parental U937 and CEM cells, respectively, in the presence and absence of IR. Data in Figure 1A illustrate Western blot for CAT, SOD, and actin. Overall, the expression of intracellular CAT in T-cells (lanes 1–4; upper panel) was notably lower than in myeloid cells (lanes 5–8; upper panel). Interestingly, IR did not affect CAT expression levels in CEM (lanes 1–2; upper panel) or U937 (lanes 5–6; upper panel), but it did have effects on ACH2 and U1 cells (lanes 3–4 and 7–8; upper panel). ACH2 cells treated with IR showed decreased CAT expression levels (lane 4; upper panel), while U1 cells showed elevated expression levels when treated with IR (lane 8; upper panel). Similarly, SOD expression was ubiquitous in uninfected cells (lanes 1, 2, 5, and 6; middle panel), except for IR-treated U937 cells where a slight decrease was noted (lane 6; middle panel). Once again, IR elicited a change in SOD expression levels in infected cells (ACH2 and U1). ACH2 cells treated with IR showed a decrease in SOD expression (lane 4; middle panel) when compared to untreated cells (lane 3; middle panel). U1 cell levels were surprisingly low (lane 7; middle panel), although when treated with IR, a minor increase in SOD expression was observed (lane 8; middle panel). Densitometry quantitation of the blots in Figure 1A shows a 2-fold difference in CAT levels between T- (lanes 1 and 2) and myeloid (lanes 3 and 4) cells (Figure 1B). SOD levels were unaltered in most cell types (lanes 1, 2, and 3), with the exception of U1 cells, with a 9-fold decrease (lane 4) when compared to U937 control cells. To confirm that innate cellular differences in CAT and SOD were responsible for the differential susceptibility to IR, exogenous CAT (two treatments) and SOD (two treatments) were added to cells treated with IR and cART (indinavir, tenofovir, emtricitabine, and lamivudine) (Figure 1C). Preliminary titration experiments using CAT and SOD enzymes on these cells and found 3.08 U and 16.82 U to be optimal dose, respectively (data not shown). Interestingly, CAT and SOD increased cell viability in U1 cells post-IR (lane 2 and 3; upper panel), while only the addition of SOD increased viability in T-cells (lane 3; lower panel).

CAT and SOD enzymes have been shown to have protective effects against IR-induced ROS. Overall, these data indicate that there are inherent differences of peroxisome-related enzymes in T- and myeloid cells, which are altered by HIV-1 and IR. Specifically, IR causes a decrease in SOD in HIV-1-infected T-cells, suggesting that SOD may play a role in T-cell susceptibility to IR. Interestingly, IR caused an increase in both CAT and SOD in HIV-1-infected myeloid cells indicating that these enzymes may be responsible for the resilience of infected myeloid cells. These findings suggest that therapeutic strategies may need to be tailored to cell types and may need to be designed to compensate for the deregulation of protective enzymes due to infection and IR.

### 3.2. Combined Treatment with IR and Mtor Inhibitor Selectively Induces Death of HIV-1-Infected T-Cells

**Figure 2:**
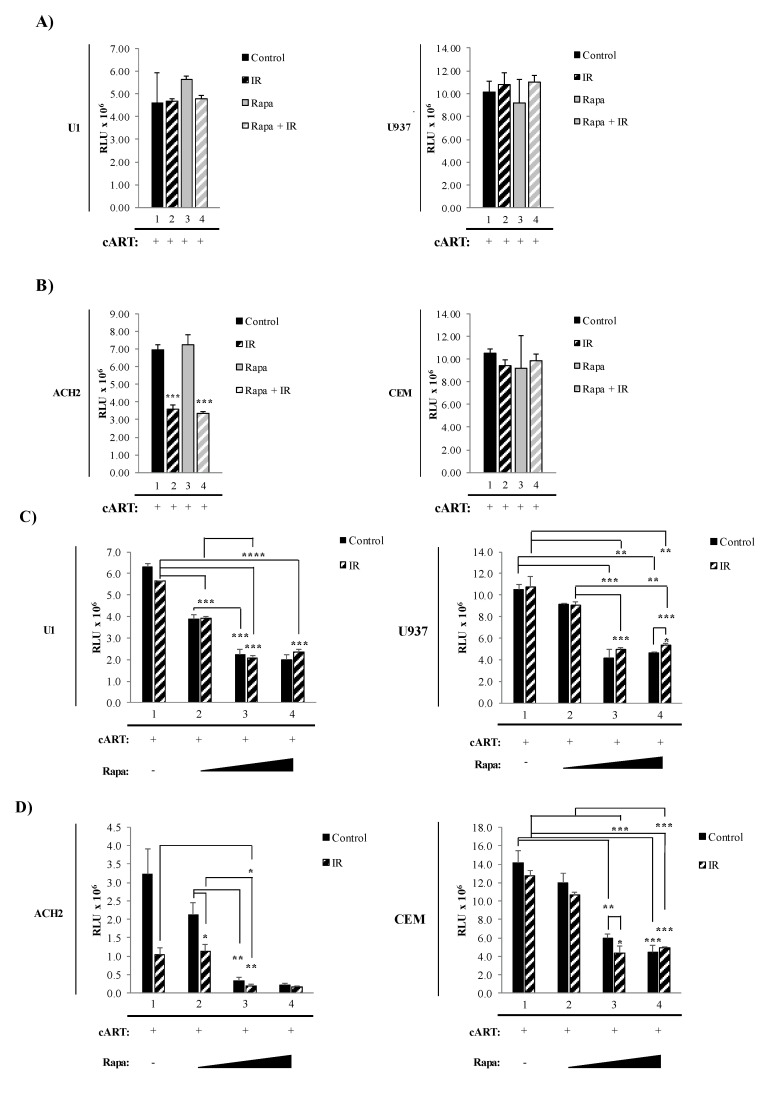
IR and autophagy inducing (AI) drugs affect the cell viability of HIV-1-infected cells. (**A**) Cell viability of HIV-1-infected monocytes cells (U1) was performed 48 h post-treatment with IR (5 Gy), Rapa (50 nM; AI), and IR/Rapa. (**B**) The same treatment was performed on HIV-1-infected T-cells (ACH2). (**C**) The therapeutic window of Rapamycin (Rapa) at 0, 15, 150, and 300 nM was assessed for U1 cells. (**D**) Additionally, the same titration was performed in ACH2 cells.

Reactivation of latent HIV-1 has been associated with increased levels of secreted pro-inflammatory molecules, such as cytokines like TNF-α and viral products, including HIV-1 proteins and HIV-1 small non-coding RNA. Many of these molecules have been found to be secreted as both free protein and in association with EVs, and have the potential to affect neighboring cells [22,24,25,120,124]. The protein mTOR is involved in numerous cellular pathways involved in the production and release of viral products and pro-inflammatory molecules, including transcription, translation, and autophagy [75,78,80,82,129]. Therefore, we hypothesized that inhibition of mTOR could potentially mitigate the LRA induction of pro-inflammatory effects by limiting the production of inflammatory molecules via inhibition of transcription/translation and release of inflammatory molecules in extracellular vesicles by induction of autophagy. To address this, we have incorporated the mTORi, Rapa, into our “shock and kill” strategy. As these alterations could have an impact on the effectiveness of IR to induce cell death selectively, we asked whether there were changes in cell death post-treatments with our novel regimen. To test this, we simultaneously exposed myeloid and T-cells to a combination of higher IR dosage and mTORC1 inhibitor (Rapa). All cells (U1, U937, ACH2, and CEM) were pretreated with indinavir, tenofovir, emtricitabine, and lamivudine to simulate the state of cART in patients. The latently infected (U1) and uninfected (U937) monocytic cell line were exposed to either 0.01% DMSO (control; lane 1), 5 Gy of IR (lane 2), Rapa (50 nM; lane 3), or combination of Rapa/IR (5 Gy/50 nM; lane 4) (Figure 2A). At 48 h post-treatment, the cells were harvested and cell viability was analyzed. Not surprisingly, U1 and U937 cell viability were unaffected by all treatments (left and right panels; Figure 2A). In contrast, the same treatment on infected ACH2 T-cells elicited a significant decrease to cell viability in response to IR (lane 2, *p*-value ≤ 0.001) and IR/Rapa (lane 4; *p*-value ≤ 0.001) treatment as compared to untreated ACH2 cells (lane 1) (left panel; Figure 2B). Furthermore, treatment with Rapa alone (lane 3) did not affect T-cell viability. To assess the specificity of IR-induced cell death observed in latently infected cells, uninfected cells (CEM) were subjected to identical treatments, which resulted in no change in viability to the treatment regimens (right panel; Figure 2B). Collectively, these data suggest that infected and uninfected myeloid cells are more resistant to cell death than T-cells. Moreover, these data suggest that the treatment with IR selectively induces cell death of infected T-cells, but not uninfected T-cells. 

In Figure 2A,B, we utilized a combination of 5 Gy of IR and 50 nM of Rapa to induce cell death in HIV-1-infected myeloid and T-cells. However, this strategy was ineffective in eliciting myeloid cell death. To address this, we increased the concentration of Rapa and utilized cell starvation to synchronize the cells to G_0_ stage of the cell cycle to minimize sample variation, promote enhanced response to IR [130,131,132], and further inhibit mTOR [133,134,135,136,137]. Data in Figure 2C,D show a titration of Rapa, on nutrient-deprived cells (3% FBS for 48 h). A significant decrease in U1 cell viability (*p*-value ≤ 0.001) at 15 nM (lane 2), 150 nM (lane 3), and 300 nM (lane 4) was observed (left panel; Figure 2C). The highest decrease in viability was between 150 nM and 300 nM yielding a 1.8 to 1.7-fold decrease, respectively. Conversely, no statistically significant viability change was observed on uninfected U937 myeloid cells (right panel; Figure 2C). Control-infected T-cells showed a significant decrease (*p*-value ≤ 0.05) in viability at 150 nM and 300 nM (Figure 2D; lanes 3 and 4, respectively; left panel). The highest viability decrease was at 300 nM Rapa (lane 4), with an 11-fold decrease compared to untreated cells (lane 1). CEM uninfected T-cells showed a statistically significant decrease (*p*-value ≤ 0.001), with a 2.3-fold decrease at 150 nM, and a 2.8-fold decrease at 300 nM Rapa. 

Altogether, these data suggest inherent differences in susceptibility to IR in different cell types with IR resilience in myeloids and IR susceptibility in T-cells. The innate resistance to IR-mediated cell death by infected myeloid cells can be reduced by introducing higher concentrations of Rapa and starvation. However, the combinatorial treatment elicited cell death in T-cells and, to a lesser extent, myeloids. The myeloid’s innate resistance to cytopathic effects suggests that the small decrease in cell viability could potentially be initial stages of cell death in myeloids. Therefore, the therapeutic strategy could be enhanced by extending the incubation period post-treatment.

### 3.3. The Combination of IR, Serum Starvation, and Mtor Inhibition Induces Cell Death in HIV-1-Infected Immune Cells

**Figure 3:**
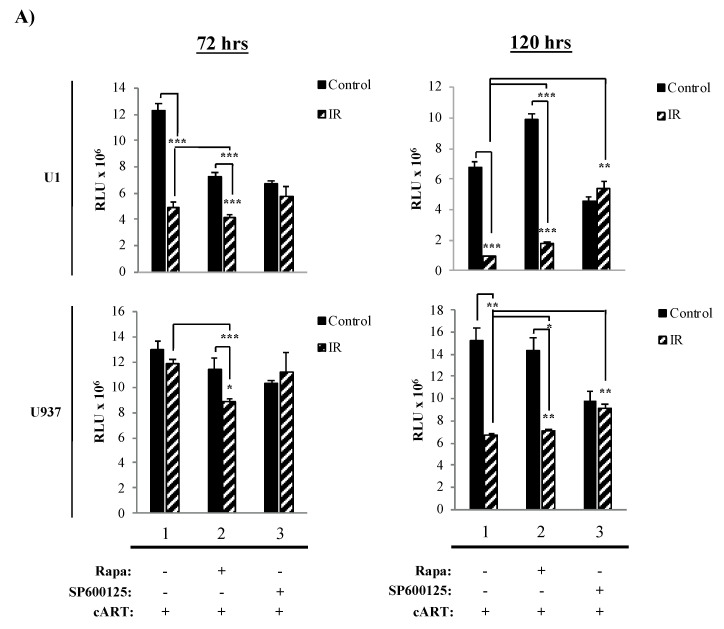

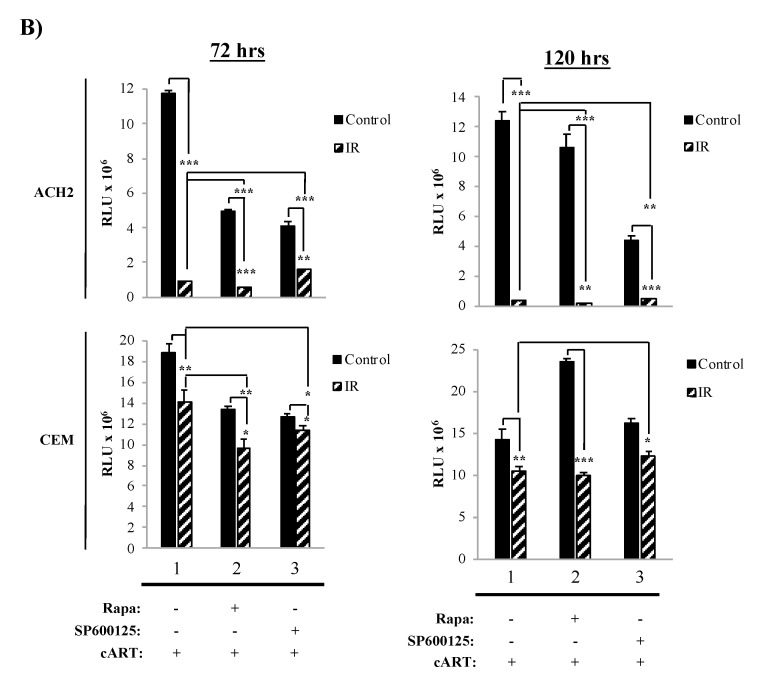
A combination of IR, AI, and serum starvation induces cell death of HIV-1-infected cells. Cell viability was assessed at two-time points (72 h and 120 h). All cells were pretreated for 3 days with cART (10 µM cocktail). Additionally, cells were treated with IR^-/+^(10 Gy), 0.1% DMSO (control), Rapa (300 nM; AI via mTOR inhibition) or SP600125 (300 nM; JNK pathway inhibitor). (**A**) Time titration for U1 and (**B**) ACH2 cells.

The data in Figure 2 showed a therapeutic dose for Rapa, where HIV-1-infected cells had the highest susceptibility to cell death and suggested that at 48 h, myeloid cells were beginning to undergo cell death. Next, we sought to increase the amount of cell death in HIV-1-infected cells and further optimize the treatment regimen. To accomplish this, we performed several changes to the treatment scheme by (1) increasing the amount of IR to 10 Gy; (2) treating cells with 300 nM Rapa; (3) inducing serum starvation by culturing cells, immediately before IR, in fresh media containing 3% FBS; and (4) increasing the length of time (72 h and 120 h) post-treatment (i.e., IR/Rapa) prior to harvesting for cell viability measurement. We additionally used SP600125, a c-Jun N-terminal kinase inhibitor, as a negative control as it does not interact with mTOR [138,139]. Data in Figure 3A demonstrate that U1 cell viability is significantly affected by IR treatment at both 72 h and 120 h (lane 1; *p*-value ≤ 0.001), and the addition of Rapa enhanced the effects of IR at 72 h (lane 2; *p*-value ≤ 0.001), while the use of SP600125 had no additional effects to IR (lane 3). However, at 120 h, U1 cells, treated with Rapa only, increased in cell viability (lane 2). The combination of IR/Rapa significantly decreased viability, but not as low as upon IR treatment only (control, lane 1), suggesting potential Rapa-induced protection at longer time points (i.e., 120 h). U937 cells were not significantly affected by Rapa treatment when evaluated at either 72 h or 120 h, but there was a significant change when cells were treated with IR/Rapa (Figure 3A; lower panels; lane 2).

Data in Figure 3B demonstrate the effects of the same treatment scheme on T-cells. Not surprisingly, at 72 h, IR alone yielded a highly significant decrease in cell viability in infected T-cells, which was enhanced at 120 h (lane 1; *p*-value ≤ 0.001). The addition of Rapa further enhanced the effects of IR, which was most noticeable at 120 h (lane 2; *p*-value ≤ 0.001). Such effects were potentially reverted by the addition of SP600125, as noted by a slight increase in cell viability (compare lane 1 to lane 3; *p*-value ≤ 0.0001 for 72 h and *p*-value ≤ 0.001 for 120 h). Uninfected cells (CEM) demonstrated increased resistance to all treatments (i.e., IR, Rapa, and/or SP600125). These data suggest that HIV-1-infected T-cells are selectively susceptible to the combined treatment of IR and Rapa, especially at longer time points after treatment. This is potentially due to the T-cell’s inability to repair cellular damage, which is more clearly manifested at later time points. Overall, these data validate the existence of a fundamental difference in resistance to IR and the downstream effects of mTORi between myeloid and T-cells, suggestive of a link between the survival of specific infected cell types and the maintenance of the latent viral reservoir. More importantly, these findings confirm that inhibition of mTOR does not significantly reduce the cytotoxic effects of IR in HIV-1-infected cells. 

### 3.4. Inhibition of mTOR Causes a Reduction in Intracellular TAR and Env RNA in HIV-1-Infected Myeloid Cells 

**Figure 4:**
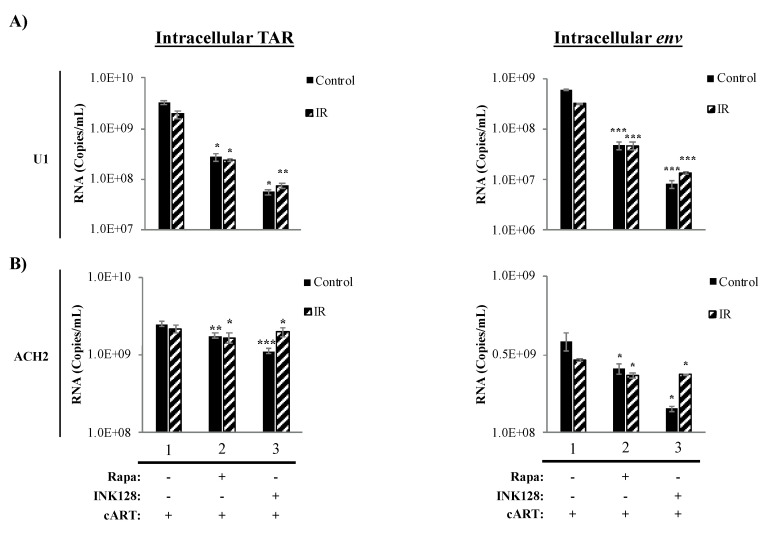
AI drugs mediate viral RNA levels in infected myeloid and T-cells. AI drugs (mTOR inhibitors; Rapa and INK128 at 300 nM) were administered as described in Figure 3, and cells were allowed to incubate for 5 days. Rapa is an mTOR complex-1 inhibitor, while INK128 is a more potent mTOR complex-1/2 inhibitor. Intracellular short RNA transcript TAR and *env* RNA was measured via RT-qPCR for U1 (**A**) and ACH2 (**B**) cells.

The protein mTOR has been shown to have effects on some cellular pathways, including transcription, translation, and autophagy. Specifically, the inhibition of mTOR has been found to suppress transcription of cellular receptors (i.e., CCR5) and the HIV-1 LTR during infection [59,75,82]. This reduction in transcription is accompanied by an overall suppression of cellular host translation, with potential inhibitory effects on viral cap-dependent mRNA translation. Further, mTOR inhibition has been found to activate autophagy during HIV-1 infection resulting in induced clearance of HIV-1 transcription factors (i.e., Tat) [59,69,98]. Moreover, we recently observed that suppressed transcriptional states of HIV-1-infected cells might further promote HIV-1 transcriptional silencing by the generation of non-coding RNA, such as TAR and TAR-*gag* (produced as a result of non-processive transcription), via mechanisms similar to 7SK, NRON, HOTAIR, and Xist RNAs [20,25]. Therefore, we explored the effects of the proposed treatment regimen on the transcription of HIV-1 TAR and env (genomic) RNA on myeloid and T-cells. Data in Figure 4 illustrate the effects of the mTORi drugs, Rapa (mTORC1 inhibitor) and INK128 (mTORC1/2 inhibitor), on transcription in HIV-1-infected monocytes (U1) and T-cells (ACH2). In infected U1 cells, we observed a 12.2-fold (*p*-value ≤ 0.05) decrease in TAR RNA levels when treating cells with Rapa and in combination with IR (Figure 4A; lane 2, left panel). U1 cells treated with INK (lane 3) decreased TAR RNA levels by 59-fold (*p*-value ≤ 0.05), and when treated with IR/INK128 it decreased by 26-fold (*p*-value ≤ 0.01). For intracellular env RNA (Figure 4A; right panel), Rapa and IR/Rapa caused a 12-fold decrease (*p*-value ≤ 0.001; lane 2) and INK128 caused a 75-fold decrease (*p*-value ≤ 0.001; lane 3), while IR/INK128 caused a 22-fold decrease (*p*-value ≤ 0.001) in RNA levels. 

Data in Figure 4B show the effects of IR and mTORi on intracellular *TAR* (left panel) and *env* RNA levels (right panel) in HIV-1-infected T-cells (ACH2). Interestingly, a significant but minimal decrease was observed for TAR RNA in ACH2 cells treated with Rapa (1.4-fold; *p*-value ≤ 0.01), and similarly with IR/Rapa (1.3-fold; *p*-value ≤ 0.001, lane 2). INK128 and IR/INK128 treatment yielded a 2.3-fold (*p*-value ≤ 0.001) and 1.1-fold (*p*-value ≤ 0.05) decrease in TAR RNA levels, respectively (lane 3). In the case of *env* RNA, Rapa alone (1.5-fold; *p*-value ≤ 0.05) and in combination with IR caused a significant decrease (1.3-fold; *p*-value ≤ 0.05, lane 2). INK128 alone caused the highest reduction in *env* RNA levels (2.7-fold; *p*-value ≤ 0.05), which was less drastic upon IR/INK128 treatment (1.3-fold; *p*-value ≤ 0.05, lane 3).

Altogether, the use of a mTORi resulted in a reduction in full-length transcription in HIV-1-infected cells. Not surprisingly, there was a difference in response to mTOR inhibition by myeloid and T-cells, potentially owing to innate differences in transcriptional machinery [140,141,142,143]. The reduction in both TAR and *env* RNA in myeloid cells suggests inhibition of transcription at the initiation level, indicative of lower RNA Polymerase II loading on to the HIV-1 LTR. Furthermore, utilization of the mTOR1/mTOR2 inhibitor INK128 elicited a greater response in myeloid cells as compared to the mTOR1 inhibitor, Rapa, suggesting both complexes play a role in transcriptional regulation of HIV-1. Conversely, mTOR inhibition predominately elicited a reduction in *env* (i.e., genomic), suggesting transcription elongation inhibition. Although mTOR inhibition caused a reduction in full-length RNA, effectively lowering the production of viral proteins, the persistence of non-processive transcription resulting in the production of TAR RNA from HIV-1-infected T-cells could potentially continue to contribute to inflammation in neighboring cells. 

### 3.5. Inhibition of mTOR Suppresses the Release of Evs Carrying TNF-α and Viral Proteins in HIV-1-Infected Myeloid Cells 

**Figure 5:**
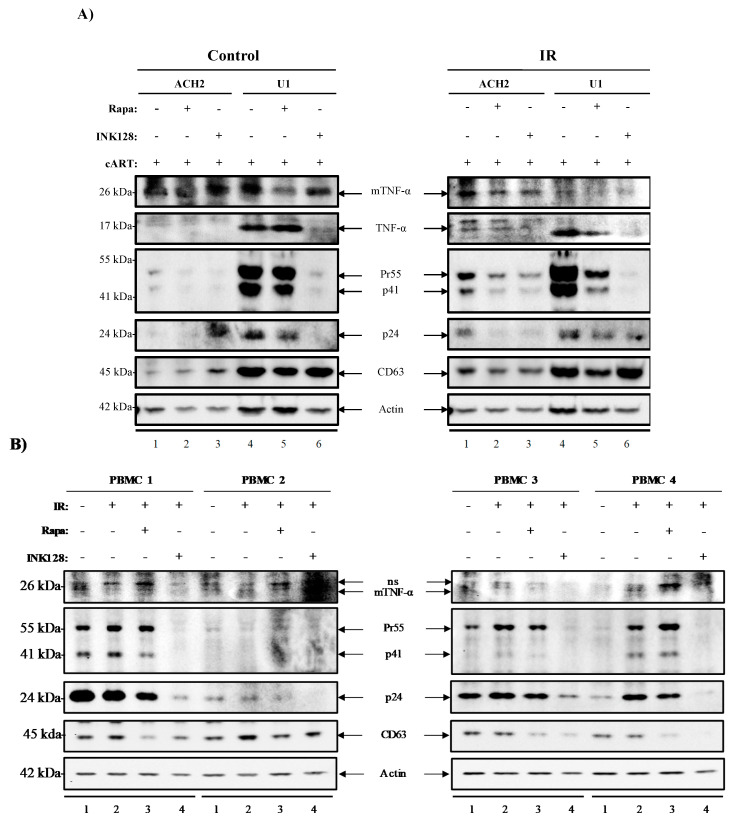
IR and AI treatment decrease Pr55/p24, CD63, and TNF-α protein expression in HIV-1-infected cells. EVs from supernatant material from cells harvested in Figure 4 were isolated with NT80/82. ACH2 and U1 cells (**A**) and PBMC patient samples (n = 4) (**B**) were Western blotted for TNF-α, Pr55/p24, CD63 and actin. Non-specific bands were denoted by “ns”.

We have previously shown that the use of various modulators (i.e., IR, mTORi, and serum starvation) may selectively decrease in cell viability of HIV-1-infected T-cells and decrease transcription in HIV-1-infected myeloid and T-cells. Furthermore, we have observed a significant decrease in viral transcription (TAR and *env* RNA) upon application of mTORi in myeloid and T-cells indicating secondary effects of the treatment. We next sought to explore the effects of our treatment in mediating (1) LRA induced EV-associated cytokine release (TNF-α or membrane-bound TNF-α), (2) translation of viral proteins (Pr55, p41, and p24), and (3) EV release. It is important to examine cytokines in association with EVs as recent findings have described a system of EV-associated pro-inflammatory cytokines, which has been shown to mediate cell-cell communication in multiple viral infections [22,24,45,101,120,122,123,144]. In Figure 5A, we examined EVs isolated by Nanotrap particles (NT80/82) from ACH2 and U1 cell supernatants. We found that Rapa alone decreased the presence of membrane-bound TNF-α (mTNF-α) in both ACH2 and U1 cells (control; lanes 2 and 5). However, INK128 had no effects on mTNF-α levels in ACH2 cells (control; lane 3), but it did decrease mTNF-α levels in U1 cells (control; lane 6). We next evaluated EVs from IR-treated cells and observed a decrease in mTNF-α levels upon Rapa and INK128 treatment for both ACH2 (IR; lanes 2 and 3) and U1 cells (IR; lanes 5 and 6). 

We next evaluated EV-associated TNF-α levels in EVs from control cells, where TNF-α was not detected in ACH2 cells (control; lanes 1–3). EVs from control U1 cells were positive for TNF-α, where Rapa did not have a noticeable effect (control; lane 5), but INK128 drastically reduced TNF-α levels (control; lane 6). IR treatment increased TNF-α levels on ACH2 cells (IR; lane 1), where Rapa (IR; lane 2), but more so INK128 decreased EV-associated TNF-α secretion (IR; lane 3). EVs from IR-treated U1 cells showed higher levels of TNF-α. However, the addition of Rapa (IR; lane 5) did not affect its expression. Conversely, INK128 had a drastic effect on decreasing free TNF-α expression in EVs from U1 cells. Interestingly, the use of Rapa (IR; lane 2 and 5) and INK128 (IR; lane 3 and 6) on cells treated with IR show similar effects in decreasing expression of mTNF-α and TNF-α. The viral proteins Pr55, p41, and p24 were also detected in EVs from ACH2 and U1 cells. When comparing control and IR-treated cells, overall viral protein secretion (Pr55, p41, and p24) was increased after IR treatment (Figure 5A; control and IR).

Both Rapa (control/IR; lane 2 and 3) and INK128 (control/IR; lane 5 and 6) decreased the levels of Pr55, p41, and p24 in EVs from ACH2 and U1 cells, but more drastically in U1 cells treated with INK128 (control/IR; lane 6). CD63 levels in EVs from ACH2 cells were increased after Rapa (control; lane 2) and more so after INK128 (control; lane 3). U1 cells showed a slight decrease in CD63 secretion when treated with Rapa (control; lane 5) and INK128 (control; lane 6). IR treatment caused a slight decline in overall CD63 levels on both cell types after treatment with Rapa or INK128. The Western blots in Figure 5A were quantitated by densitometry analysis and can be found in the Supplementary Material section as Figure S1. Additionally, repeats of this experiment were performed in biological triplicates of U1 and ACH2 to validate our observations (Figure S2A–C), suggesting that the combination of IR/INK128 (lane 4) is the most potent combination for U1 cells. This was evident due to the statistically significant decreases in mTNF-α levels, and almost completely abolishing Pr55 and p24 levels (Figure S2B). On the other hand, IR alone in ACH2 cells was sufficient to abolish mTNF-α and Pr55 levels, potentially due to significant decreases in cell viability (Figure S2C). 

Altogether, these data suggest that treatment with mTORi (Rapa and INK128) not only causes a decrease in EV-associated TNF-α, but also of Pr55, p41, and p24 in EVs from HIV-1-infected cells. Interestingly, the application of these treatments has little to no effect on the release of vesicles, as estimated by CD63 levels, suggesting there are alterations in the packaging of these products rather than an overall decrease in the number of vesicles released. These findings are in line with an intracellular reduction in transcription/translation and induction of autophagy.

### 3.6. Inhibition of mTOR Suppresses TNF-α and Viral Protein Expression in Evs from Latently HIV-1-Infected Primary Cells

Next, we attempted to validate our observations regarding the use of IR, serum starvation, and mTORi on HIV-1-infected peripheral blood mononuclear cells (PBMC) from individuals under cART [127,128]. Data in Figure 5B show EVs enriched from culture supernatants from PBMCs 1–4 (n = 4) cultured for 11 days and treated with IR (0.5 Gy), Rapa (50 nM), or INK (50 nM). Following enrichment, EVs were analyzed using Western blot analysis for the presence of mTNF-α, Pr55, p41, p24, and CD63. IR treatment (lanes 2) caused an increase in pro-inflammatory cytokine (mTNF-α) on PBMCs 2, 3, and 4. However, PBMC 1 displayed a slight decrease. Viral proteins Pr55/p41 were increased upon IR in PBMCs 1, 3, and 4. However, levels in PBMC 2 were slightly decreased. HIV-1 p24 levels were slightly decreased in PBMC 1; however, they were unchanged in PBMC 2 and increased in PBMCs 3 and 4 (lane 2). The EV marker, CD63, was detected in all samples, with IR inducing slight increases in CD63 were noted on EVs from PBMC 1 and 2 and a slight decrease in PBMC 3 and 4. As expected, Rapa treatment (lanes 3) showed a slight decrease in mTNF-α from PBMC 2 and 3, but not from PBMC 1 and 4. However, the more potent mTOR inhibitor, INK128 (lanes 4), caused a drastic decrease in mTNF-α levels across all patients. Interestingly, a similar trend was observed for expression levels of viral proteins. Rapa caused a slight decrease in EV-associated Pr55, p41, and p24 across all PBMCs. Although, INK128 treatment presented a stronger decrease in EV-associated Pr55, p41, and p24 in all four PBMCs. Surprisingly, despite the overall reduction in pro-inflammatory cytokine and viral proteins, Rapa and INK128 also decreased expression of CD63 in all patient PBMCs when compared to IR treatment alone (lanes 3 and 4 vs. 2). Actin protein levels remained similar across all lanes, with a slight decrease observed for INK128 treatment. Densitometry analysis (Figure S3) revealed that, once again, the combination of IR/INK128 was the most potent at decreasing cytokine levels (i.e., mTNF) and viral proteins (i.e., Pr55, p41, and p24), and that this was also true for PBMCs.

Overall, these data suggest a more robust effect of INK128, when compared to Rapa, in decreasing the IR-mediated expression of mTNF-α, Pr55, p41, and p24 associated with EVs, potentially due its dual inhibitory effect against mTORC1/2 further affecting translation, transcription, and autophagy. 

### 3.7. mTORi Drugs Mitigate TNF-α Mediated HIV-1 Promoter Activation 

We have observed that mTORi drugs have suppressive effects on the release of EV-associated TNF-α and viral proteins (Pr55, p41, and p24). We also observed the downregulation of the EV-associated CD63 when using inhibitors against mTORC1 (Rapa) or mTORC1/2 (INK128) from IR-treated PBMCs. We next evaluated the inhibitory effects of Rapa/INK128 by using the PBMC EVs (similar to Figure 5B) on an inducible HIV-1-infected HeLa cell with a triple mutation in the Tat gene (HLM-1). These cells contain a mutant HIV-1 provirus that upon co-cultivation with Tat or stimuli, such as TNF-α, PMA, or sodium butyrate, HLM-1 cells express full-length viral transcripts [145,146]. Given that HIV-1 latency reactivation is associated with increased production of viral proteins and expression of pro-inflammatory cytokines, we hypothesized that if Rapa or INK128 effectively mitigates EV-associated cytokine and viral protein release from reactivated cells (i.e., via IR), the EVs isolated from Rapa or INK128-treated infected PBMCs would not induce expression of HIV-1 in HLM-1 cells. 

**Figure 6:**
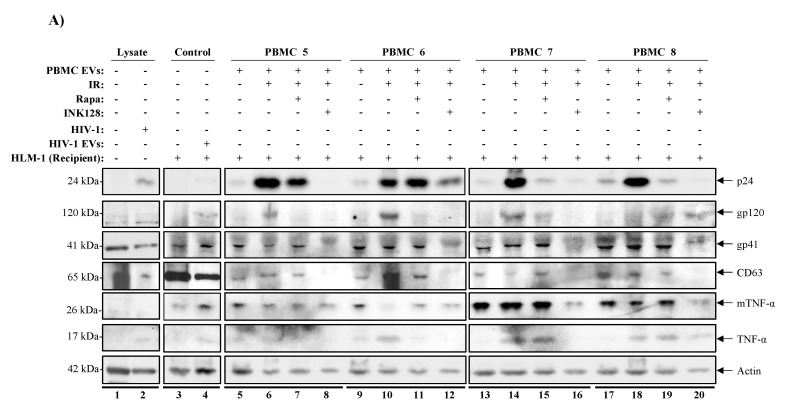

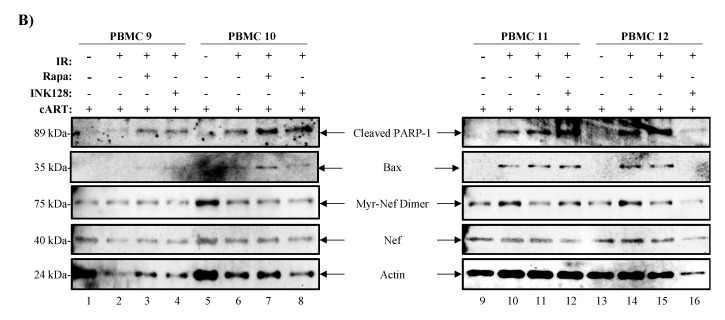
mTORi drugs mitigate the effects of EV-TNF-α and viral proteins on recipient cells. (**A**) Nanotraped EVs (100 μL; NT80/82) from four PBMCs treated with control (untreated), IR (10 Gy), IR/Rapa (10 Gy/300 nM), and IR/INK128 (10 Gy/300 nM) were cultured with HLM-1 recipient cells which contain a triple mutation in Tat and transcription can be induced by IR and TNF-α. Western Blot analysis was used to detect the presence of viral proteins (i.e., p24, gp120, gp41), EVs marker (i.e., CD63), cytokine (i.e., mTNF-α and TNF-α), and actin. (**B**) Cell pellets from PBMCs 9–12 infected with HIV-1 89.6 were treated with IR (0.5 Gy), Rapa (50 nM) or INK128 (50 nM), and 3% FBS media for 5 days prior to Western Blot analysis for markers of cell death (i.e., PARP-1 and Bax) and virus (i.e., Nef).

Therefore, supernatant material containing EVs from donor PBMCs (5, 6, 7, and 8) untreated or treated with IR, IR/Rapa, or IR/INK128 were incubated with recipient HLM-1 cells for 72 h. Additionally, the viral infectivity enhancer, “Infectin” (a generous gift from Dr. Yuntao Wu), was also added to all samples prior to incubation. After incubation, supernatant material from recipient HLM-1 cells (treated with supernatants from PBMCs 5, 6, 7, and 8) were collected and separated from cell pellets and enriched for EVs using Nanotrap particles (NT80/82/86) and analyzed by Western blot. Analysis of HLM-1 cells showed that IRed donor PBMC supernatants caused an increase in EV-associated p24 abundance in all recipient HLM-1 (lanes 6, 10, 14, and 18) compared to untreated controls (lanes 5, 9, 13, and 17; Figure 6A). Interestingly, supernatants from IR/Rapa-treated PBMCs caused an overall decrease in p24 level (lanes 7, 15, and 19). However, supernatants from IR/INK128-treated PBMCs completely abolished p24 levels in 3 out of 4 recipient cells (lanes 8, 12, and 20). PBMC 6 was less responsive to treatments, as evidenced by a less potent effect of both Rapa (lane 11) and INK128 (lanes 12). Other viral proteins (i.e., gp120 and gp41), exosomal marker (i.e., CD63), and cytokines (i.e., TNF-α) were also assessed. We observed that EVs from IRed donor PBMCs consistently increased protein levels for gp120, CD63, and TNF-α in recipient cells, which were reduced after Rapa addition and most efficiently reduced after of INK128 addition. Protein levels of gp41 and mTNF-α were less responsive to IR and IR/Rapa donor EVs. However, IR/INK128 was still able to decrease protein levels on all PBMCs. Overall, these data suggest that mTORC1 inhibition (Rapa) has mild and varied effects in mitigating TNF-α release from HIV-1-infected PBMCs. However, inhibition of mTORC1/2 (INK128) has a more robust effect in reducing TNF-α release, and additionally p24, gp120, gp41, and CD63, as evidenced by a consistent decrease in protein expression in all four PBMCs. Finally, densitometry analysis of these blots was performed (Figure S4) and we can observe that the EVs released from infected PBMCs treated with IR (lane 2) contained significantly higher levels of TNF-α causing the indicator cell line, HLM-1, to produce higher levels of p24, gp120, and gp41. Interestingly, the EVs from infected PBMCs treated with the combination of IR/INK128 had a significantly reduced ability to activate HLM-1 cells, as evidenced by the decreased levels of p24 and mTNF-α, and the absence of gp120, gp41, and TNF-α. These data suggest that the combination of IR/INK128 allows for activation of the latent virus, while also suppressing the release of cytokines, minimizing the side effects associated with LRAs.

In order to validate our observations regarding the effects of our treatment scheme on decreasing the viability of HIV-1-infected cells, we infected four healthy PBMCs (9-12) with dual-tropic HIV-1 isolate 89.6 and promoted latency by use of IL-7 and cART for 6 days prior to our treatment regimen (i.e., IR, mTORi, and starvation), as described previously. The cell pellets were harvested, lysed, protein levels quantified (12.5 µg loaded per lane) and analyzed, using Western blot, for the presence of markers of cell death (i.e., Poly [ADP-ribose] polymerase 1 (PARP-1) and Bcl-2-associated X protein (Bax)) that would suggest decreased cell viability, and virus (i.e., Nef) to validate for productive viral infection. In Figure 6B, cleaved PARP-1 levels increased in 3/4 PBMCs when comparing untreated (lanes 1, 5, 9, and 13) to IR-treated cells (lanes 6, 10, and 14). Interestingly, treatment with IR/Rapa increased the levels of cleaved PARP-1 in 4/4 PBMCs (lanes 3, 7, 11, and 15) when compared to untreated or IR only treated cells. Treatment with IR/INK128 also showed an increase in 3/4 PBMCs (lanes 4, 8, and 12), similar to IR/Rapa; however, a decrease was noted in 1/4 PBMCs (lane 16). We next probed for the apoptotic effector protein Bax and found that IR alone significantly increased Bax levels in 3/4 PBMCs (lanes 6, 10, and 14). However, IR/Rapa increased Bax levels in 4/4 PBMCs (lanes 3, 7, 11, and 15), while IR/INK128 increased the levels in 3/4 PBMCs (lanes 4, 8, and 12). The viral protein Nef was present in all lanes. However, two versions of Nef were detected, a myristylated viral protein Nef homodimer (Myr-Nef Dimer) and an unmodified Nef. Myr-Nef Dimer was detected at varying levels across the 4 PBMCs. In 2/4 PBMCs, IR increased Myr-Nef Dimer levels (lanes 10 and 14) compared to control (lanes 9 and 13, respectively) and the addition of Rapa (lanes 11 and 15) or INK128 (lanes 12 and 16) caused a decrease in Myr-Nef Dimer levels. The unmodified Nef was detected at relatively constant levels across all treatments; however, slightly decreased levels were detected in 4/4 PBMCs treated with IR/INK128 (lanes 4, 8, 12, and 16). Finally, Actin levels were decreased in 2/4 PBMCs when treated with IR only (lanes 2 and 6), 2/4 PBMCs when treated with IR/Rapa (lanes 3 and 7), and 3/4 PBMCs when treated with IR/INK128 (lanes 4, 8, and 16). Densitometry analysis of these blots can be found in Figure S5. 

Altogether, these data suggest that IR, and more so IR/Rapa or IR/INK128, increases cell markers of cell death as evidenced by the increased levels of cell death markers cleaved PARP-1 and Bax, and decreased levels of Actin. The data also suggest that our regimen decreases HIV-1 protein levels, especially when using a combination of IR and mTORi. 

## 4. Discussion

Current approaches to target HIV-1 reservoirs use the “shock and kill” strategy to activate the latent virus and kill infected cells via immune cell targeting. However, this strategy has limited success at depleting HIV-1 reservoirs and may cause hyper-activation of the immune system with adverse systemic effects due to increased pro-inflammatory cytokine release (i.e., TNF-α). For this reason, we explored the use of a treatment scheme that uses cART to control viral spread, IR to activate the virus, and mTORi (i.e., serum starvation and Rapa or INK128) to mitigate TNF-α release. This regimen aims to more broadly target the HIV-1 reservoir since IR crosses potential anatomical barriers. At the same time, we observed that HIV-1-infected cells are more susceptible to IR than uninfected cells, pointing towards a repair mechanism that may be aberrant in the infected cells. A potential player in cell resilience may be the levels of peroxisome-related enzymes, CAT and SOD, which seem to differ based on cell type (i.e., myeloid and T-cell) and infection status. It was also important to investigate the effects of viral activation on EV cargo packaging and secretion since EVs can transport cytokines and viral proteins to neighboring recipient cells and induce functional effects (i.e., activation of transcription, inflammation, or cell-cell syncytia). The proposed treatment scheme potentially addresses the current concern regarding “shock and kill” therapies, by effectively activating latent HIV-1 in reservoirs, preventing viral spread, inducing cell death in infected cells, and mitigating adverse side effects on recipient neighboring cells by control over EVs, cytokines, and viral proteins. Next, we will further elaborate on the potential mechanisms governing the observed effects of the proposed treatment scheme. 

In this manuscript, we unveiled central differences between myeloid and T-cell responses to IR (i.e., stress signal and inducer of ROS) [31,32,147], which may be further affected by HIV-1. Myeloid and T-cells have the potential to become infected with HIV-1, and due to the resilience of the myeloid cell types [148,149,150], potential long-term viral reservoirs may be established [54,151]. Therefore, understanding intracellular factors (i.e., peroxisome-related enzymes; CAT and SOD) that may protect infected cells against IR-induced cell death is essential to develop an effective treatment scheme, especially since CAT and SOD have already been shown to be important for cell survival via the mediation of ROS [112,113]. We found that infected T-cells contained inherently lower levels of peroxisome-related enzymes, which were further reduced after IR, especially for SOD (Figure 1A,B). Infected myeloid cells had almost no SOD. These data correlate with studies that showed that lower levels of SOD in rats resulted in significantly reduced spleen sizes, reduced number of T-cells and macrophages (among others), increases in oxidative stress in splenic tissues, and shorter lifespans [152]. Additionally, the importance of peroxisome-related enzymes to cell survival was validated by the addition of exogenous CAT/SOD, showing that CAT rescues viability in myeloid cells only, SOD rescues viability in both myeloid and T-cells (Figure 1C). 

It is also important to evaluate further the observation regarding a slight increase in CAT/SOD levels after IR on U1 cells and that U1 cells were the most resistant to cell death by IR and mTORi (Figure 2) and IR, serum starvation, and mTORi (Figure 3). We have previously demonstrated that radiotherapy-relevant doses of IR selectively induced cell death in HIV-1-infected T-cells, while infected myeloid cells have characteristic robustness to the same treatment [4]. Therefore, it may be possible that upon IR, myeloid cells increase transcription and translation of CAT/SOD, which T-cells are not able to do efficiently, conveying enzymatic protection against IR-induced ROS and promoting increased cell survival. These data correlate with previously published data regarding the differential effects of ROS on myeloid and T-cells, where myeloid cells utilize ROS signals to enhance transcription via transcription factors (i.e., NF-κB, Bach1, Nrf2, AP-1, and HIF-1alpha) promoting overall survival and T-cells undergo apoptosis [153]. Additionally, NF-κB has been shown to regulate basal and induced expression of SOD RNA [154]. Ultimately, these downstream effects of ROS on myeloid cells have been shown to protect against TNF-mediated cell death [155]. 

ROS has been shown to be important in the modulation of pathogenesis and deregulation of normal hematopoiesis by promoting genetic instability [156]; therefore, the accumulation of ROS due to lack of CAT/SOD (i.e., T-cells) could further advance cellular damage and death. Additionally, environments with low CAT/SOD and high ROS levels may result in the partial reduction in ROS and in the production of reactive nitrogen species (RNS) [157]. This is important because the overall lower levels of CAT/SOD in T-cells may result in the greater accumulation of ROS and RNS, which in combination with the IR-induced DNA damage, could explain the inherently lower T-cell viability. Future research should also consider the accumulation of RNS to fully understand the implications of low-levels of peroxisome-related enzymes on the mechanism of induced myeloid and T-cell death. 

We recently mentioned that ROS causes differences in cell fate via the activation of transcriptional factors that mediate cell survival in myeloid cells. ROS leads T-cells towards apoptosis [153], which supports our observations of decreased T-cell viability in HIV-1-infected T-cells (Figure 1, Figure 2 and Figure 3). However, it is important to explore that, despite increased T-cell death, we also observed cell-type differences in HIV-1 transcription (i.e., TAR and *env* RNA) potentially due to innate differences in transcriptional machinery [140,141,142,143] (Figure 4). The effects of our regimen (i.e., cART, IR, serum starvation, and mTORi) resulted in an overall reduction in full-length transcription of HIV-1 (*env* RNA) on myeloid and T-cells, but a reduction in non-processive transcription (TAR RNA) occurred only in myeloid cells, with more potent effects elicited by INK128. This potentially suggests that Rapa (mTORC1 inhibitor) and INK128 (mTORC1/2 inhibitor) affect cell types differentially by reducing the loading of RNA Polymerase II onto the HIV-1 LTR in myeloid cells. This will result in a decrease in both TAR and *env* RNA. However, in order to maintain levels of TAR RNA, such as in T-cells, mTORi would have to allow binding of RNA Polymerase II onto the HIV-1 LTR and only affect elongation, via mechanisms such as inhibition of RNA Polymerase II phosphorylation, mediated by CDK9 in the P-TEFb complex (i.e., CDK9/CyclinT1/Tat) which is recruited by the snRNA known as 7SK/Hexim-1. The inhibition of phosphorylation would prevent elongation and promote abortive RNA Polymerase II transcription and accumulation of TAR RNA. However, it is important to consider that the HIV-1 TAR RNA sequence is a product of transcription termination between position 55-59 of the LTR region, typically occurring in the absence of HIV-1 Tat [63,64,65,66,158,159,160]. 

Altogether, the decrease in TAR and *env* RNA levels may also suggest that mTOR inhibitors may repress the initiation of HIV-1 transcription by inhibiting the translation of HIV-1 Tat, which has not been investigated in this study. An alternative reason for non-processive HIV-1 transcription could be the clearance of HIV-1 Tat by autophagy, especially since mTORi has been shown to induce autophagy. The accumulation of TAR RNA from HIV-1-infected T-cells could potentially continue to contribute to inflammation in neighboring cells [24]. Therefore a novel treatment scheme could include the use of a transcription inhibitor, such as F07#13 [20,21,25,74]. F07#13 has shown to have suppressive effects on HIV-1 transcription, and enhanced CRISPR-cas9 gene editing, by the generation of non-coding RNAs (i.e., TAR-*gag* RNA) which act as “RNA machines” complexed with proteins such as PRC2 (involved in histone methylation), Cul4B (involved in Tat degradation), and Sin3A (involved in chromatin compaction). The addition of a transcription inhibitor along with IR and mTORi may be sufficient to protect against secretion of EVs, while further deregulating homeostasis in the infected cell, resulting in higher levels of apoptosis, especially important in HIV-1-infected myeloid cells.

Interestingly, our treatment regimen also had effects on EV release and potential packaging of cytokine and viral proteins. After analysis of EVs released by HIV-1-infected cells, we were able to deduce that in fact, IR activates HIV-1 transcription and translation, as evidenced by higher Pr55 levels in infected cell lines (Figure 5A) and primary patient cells (Figure 5B). Additionally, IR only partially increases EV-associated TNF-α levels in ACH2 and U1 cells, but EV-associated mTNF-α levels were increased in 3 out of 4 PBMCs. It was noted that PBMC 1 was the less responsive sample, which was isolated from an individual with a history consuming drugs of abuse (marijuana and alcohol). This may suggest that drugs of abuse may hinder the potential therapeutic effects of IR/mTORi/starvation. Additionally, it is important to consider that cell subpopulation present in PBMCs, before and after culture, may differ from one individual to another. Further purification of subpopulations may allow for increased consistency of results, especially since myeloid and T-cells respond differently the proposed treatment scheme. The findings suggest that use of mTORi on infected cells may potentially reduce cytopathic effects of IR and viral activation on infected cells by reducing viral and cytokine levels in EVs. The compounded effect of serum starvation and mTORi on infected cells may be enough to activate autophagy and degradation of viral proteins, such as Pr55, p41, and p24. However, it may also be sufficient to inhibit translation (~90%) of cytokines and viral proteins, which is an added effect to the repression of full-length transcription by mTORi (~10%). This is especially important since TNF-α and viral proteins have previously been shown to be associated with EVs from infected cells [116,120,161], and these data point towards potential regulatory functions of IR, serum starvation, and mTORi on EV cargo.

Rapa and INK128 not only mitigated TNF-α levels but also decreased Pr55 levels in cell lines (U1 and ACH2) and PBMCs. When further investigating the effects of latency reactivation (i.e., IR) and autophagy induction (AI; Rapa and INK128), it became evident that simultaneous inhibition of mTORC1/2 (i.e., INK128) had the strongest mitigating effects against the expression of viral proteins and cytokines in EVs from donor cells (Figure 6). We observed that latency reactivated donor cells (i.e., IR) contain higher levels of viral proteins, EVs, and cytokines and that the mTORC1 inhibitor (Rapa) can partially suppress the secondary effects of reactivation on neighboring recipient cells. However, the mTORC1/2 inhibitor INK128 has the potential to almost completely suppress secondary effects on neighboring cells, as evidenced by the absence of p24, gp120, gp41, CD64, and TNF-α from recipient HLM-1 cells. It is important to note that PBMC 6 was the only one that yielded EVs that even after treatment with INK128 was still able to activate the HLM-1 recipient cell. Interestingly, this PBMC was the only isolated from an individual with a history of 14 different prescriptions drug use, which may have resulted in cells less responsive to mTORi and activation of autophagy. Overall, these findings support the hypothesis that the induction of autophagy in infected cells may allow for the selective suppression of viral proteins and cytokines that result from viral activation. 

Myeloid cells seem to be highly responsive to targeting with mTORC1/2 inhibitors, especially in acute myeloid leukemia, where anti-tumor activity has been described [162,163,164], but also suppression of HIV-1 transcription [59]. Here we have reported that U1 cells respond well to treatment with INK128, resulting in strong suppression of EVs carrying cytokines or viral particles and slightly less suppression in T-cells (Figure 5A). Fundamental differences may exist in mTOR signaling between myeloid and T-cells, where inhibition in myeloids may lead to engaging autophagy more easily. In T-cells, mTORi has been reported to result in immunosuppression [133]. Therefore, T-cells may focus on processing signals that regulate immune responses, taking away from its ability to activate autophagy readily. As discussed previously, activation of autophagy may have a direct link to the degradation of cargo in EVs and reduction in viral protein and cytokines.

**Figure 7:**
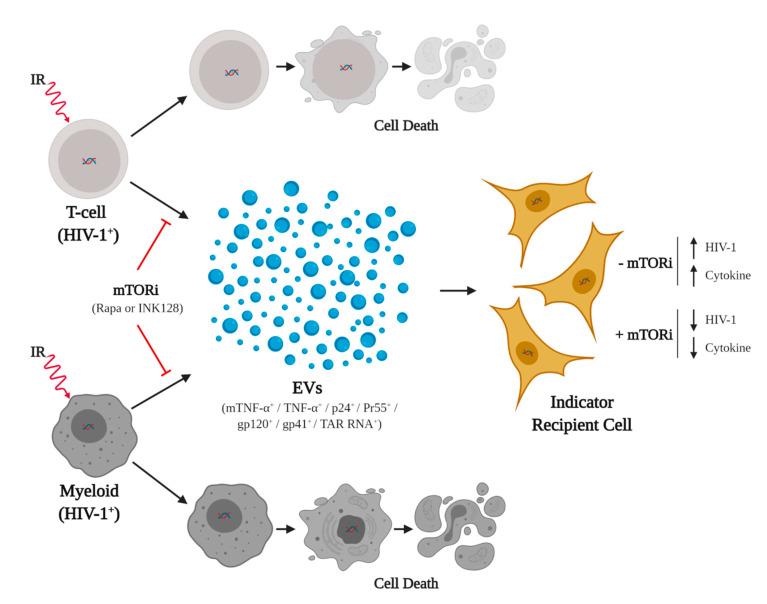
Combination of cART, Starvation, IR, and mTORi drugs suppress the effects of TNF-α and viral proteins on recipient cells. The HIV-1-infected myeloid and T-cells, located in latent reservoirs, persist despite cART pretreatment. Ionizing radiation (IR) activates HIV-1 and induces cellular stress, which leads to cell death, with increased resilience in myeloid cells. The infected cells also secrete EVs associated with pro-inflammatory cytokines (i.e., mTNF-α and TNF-α) and viral molecules (i.e., p24, Pr55, gp120, gp41, and TAR RNA) with adverse effects on neighboring recipient cells. Drugs that inhibit mTOR (i.e., Rapa and INK128), along with serum starvation, suppress the release of cytokines and viral molecules, preventing LRA related side effects. Figure created with BioRender.com.

Here, we show that a potential treatment strategy may be to selectively reactivate latent HIV-1 via the treatment with an effective stress inducer and induce death of HIV-1-infected cells, followed by combination with a mTORi drug (i.e., INK128) resulting in mitigation of adverse effects from viral proteins and cytokines (Figure 7). Rapa and more so INK128 may suppress mostly translation and/or transcription and activate autophagy in infected cells. Altogether, this treatment may help the host cell to degrade viral proteins and cytokines. This would be consistent with the findings that HIV-1 Nef, have direct inhibitory effects against Beclin-1, which is important for the formation of the autophagosome via conversion of LC3-I to LC3-II and packing of proteins. Basal levels of autophagy may promote the formation of an autophagosome; however, these may not be degraded due to inhibition of fusion with a lysosome. By consequence, HIV-1 proteins may exit the cell by secretion in EVs by exocytosis, autophagosomes, or EVs from the Endosomal Sorting Complex Required for Transport (ESCRT)-dependent pathway. Some potential explanations for the observed responses of HIV-1-infected cells to our treatments are that the viral activation by IR may cause an excess of viral proteins above what basal autophagy can take care of, leading to cellular toxicity, increased secretion in EVs, recognition by immune cells, and cell death. The increase in viral transcription, accumulation of viral products, and induced autophagy may also allow secretion of proteins and cytokines into the extracellular medium via EVs. Fortunately, the spread of HIV-1 after reactivation can be controlled by cART, and future in vivo studies (i.e., use of humanized mouse models) can determine the effects of a combination with mTORC1/2 inhibitors (i.e., INK128) to suppress side effects of reactivation related to cytokines and other proteins in EVs.

Radiation therapy (RT) has been widely and successfully used to treat many types of cancer, and in the past decades, the field has experienced major engineering and computational improvements that allow for more precise and powerful RT [31]. The techniques currently used in clinic, enable the delivery of radiation with higher precision to the desired target, minimizing side effects and damage to healthy tissues [165]. Considering the advances in RT technology, the use of IR has the potential to serve more effectively as an LRA in animal and clinical studies. Despite these advances, high radiation doses may induce multiple negative effects, such as killing of normal tissues via production of ROS and induction of double-strand DNA breaks, changes in the expression of inflammatory cytokines, chemokines, and fibrotic cytokines that result in altered cell–cell interactions and acute and chronic inflammation [166,167], the proposed treatment scheme utilizes an IR dose (0.5 Gy) that is lower than those used in cancer radiotherapy and induces minimal side effects [168,169]. Therefore, future experiments could focus on developing a therapeutic strategy for IR that, for instance, uses either low total body irradiation doses or higher local doses for strategically targeted HIV-1 reservoirs. IR is already used to suppress the immune system of the NOD/Shi-scid/IL-2Rgc null (NOG) mice to humanize them by the addition of human stem cells [170]. We have used this animal model extensively in the study of HIV-1 and HTLV-1 and observed no to minimal side effects to irradiation [4,45]. Therefore, the use of IR in our treatment regimen is applicable to the study of humanized mouse models. 

Overall, our treatment scheme uses low IR doses to initially reactivate HIV-1, while also inducing double-stranded DNA damage and p53-dependent cell death (augmented by Tat) [4,47], from which infected cells struggle to recover. Uninfected cells may more easily undergo DNA damage repair. The subsequent use of nutrient starvation may potentially push infected cells further towards death, especially since autophagy, which can provide some energy to the cell, is impaired in HIV-1-infected cells. At this stage, HIV-1-infected cells may have already committed to cell death. Use of mTOR inhibitors, such as Rapa or INK128, may potentially be able to activate autophagy in uninfected cells, increasing the chances for survival, while HIV-1-infected cells struggle. However, the pharmacological activation of autophagy in HIV-1-infected cells potentially allows for the clearing of major cytokines and viral proteins, decreasing the side effects associated with viral activation and inflammation. Interestingly, the observed suppressive effects on gene expression and translation by mTOR inhibitors is secondary to the autophagy activation in this scenario, and potentially engage after the cell has already committed towards death. 

The goal of the proposed treatment strategy is to lay a foundation for future animal and clinical studies to evaluate the potential reactivation and depletion of viral reservoirs across the body, while minimizing side effects, and effectively eradicating HIV-1 in an infected individual.

## Figures and Tables

**Table 1:** Human cohort information.

ID	Sex	Sample Type	HIV-1	cART; Years	Hepatitis B	Hepatitis C	Collection Date
1	Female	PBMC	+^*2^	+; n/a	-	-	13 September 2017
2	Male	PBMC	+^#8^	+; n/a	-	-	20 September 2017
3	Female	PBMC	+^#5^	+; 11.7	-	+	4 October2017
4	Female	PBMC	+	+; 14.9	-	-	8 November2017
5	Female	PBMC	+	+; 9.4	-	-	10 January 2018
6	Male	PBMC	+^#14^	+; 12	-	-	12 February 2018
7	Male	PBMC	+	+; n/a	-	+	1 March2018
8	Female	PBMC	+^#3^	+; 10.3	-	+	1 March 2018

Number after “#” indicates quantity of different prescription drugs or “*” drugs of abuse regularly used in addition to cART.

**Table 2:** Healthy PBMCs for infection with dual-tropic 89.6 HIV-1 and induction of latency.

ID	Lot Number	Sex	Age	Ethnicity	Sample Type	Collection Date
9	2010113879	Male	26	African American	PBMC	4 September 2019
10	2010113875	Male	26	Hispanic/Latino	PBMC	28 August 2019
11	2010113854	Female	42	Caucasian	PBMC	26 July 2019
12	2010113876	Female	43	Caucasian	PBMC	27 August 2019

## Supplementary Materials

The following are available online at https://www.mdpi.com/1999-4915/12/8/885/s1, Figure S1: Quantitation of ACH2 and U1 Western blots for Figure 5A, Figure S2: Validation of the effects of IR and mTORi on HIV-1 infected myeloid and T-cells, Figure S3: Quantitation of PBMC Western blots for Figure 5B, Figure S4: Densitometry Analysis of Western blots for EVs from PBMCs 5-8 on HLM-1 cells from Figure 6A, Figure S5: Densitometry Analysis of Western blots for PBMCs 9-12 in Figure 6B.
