# Supplementary material for: Low-Level Ionizing Radiation Induces Selective Killing of HIV-1-Infected Cells with Reversal of Cytokine Induction Using mTOR Inhibitors
Source: Viruses. 2020 Aug 13;12(8):885. doi: 10.3390/v12080885 (PMC7472203; doi:10.3390/v12080885)
Supplement: Supplementary file 1 [file viruses-12-00885-s001.pdf]

Low-Level Ionizing Radiation Induces Selective Killing of HIV-1-Infected Cells with Reversal of Cytokine Induction Using mTOR Inhibitors

Daniel O. Pinto[1], Catherine DeMarino[1], Thy T. Vo[1], Maria Cowen[1], Yuriy Kim[1], Michelle L. Pleet[1], Robert A. Barclay[1], Nicole Noren Hooten[2], Michele K. Evans[2], Alonso Heredia[3], Elena V. Batrakova[4], Sergey Iordanskiy[5], and Fatah Kashanchi*[1]

Supplementary Materials:

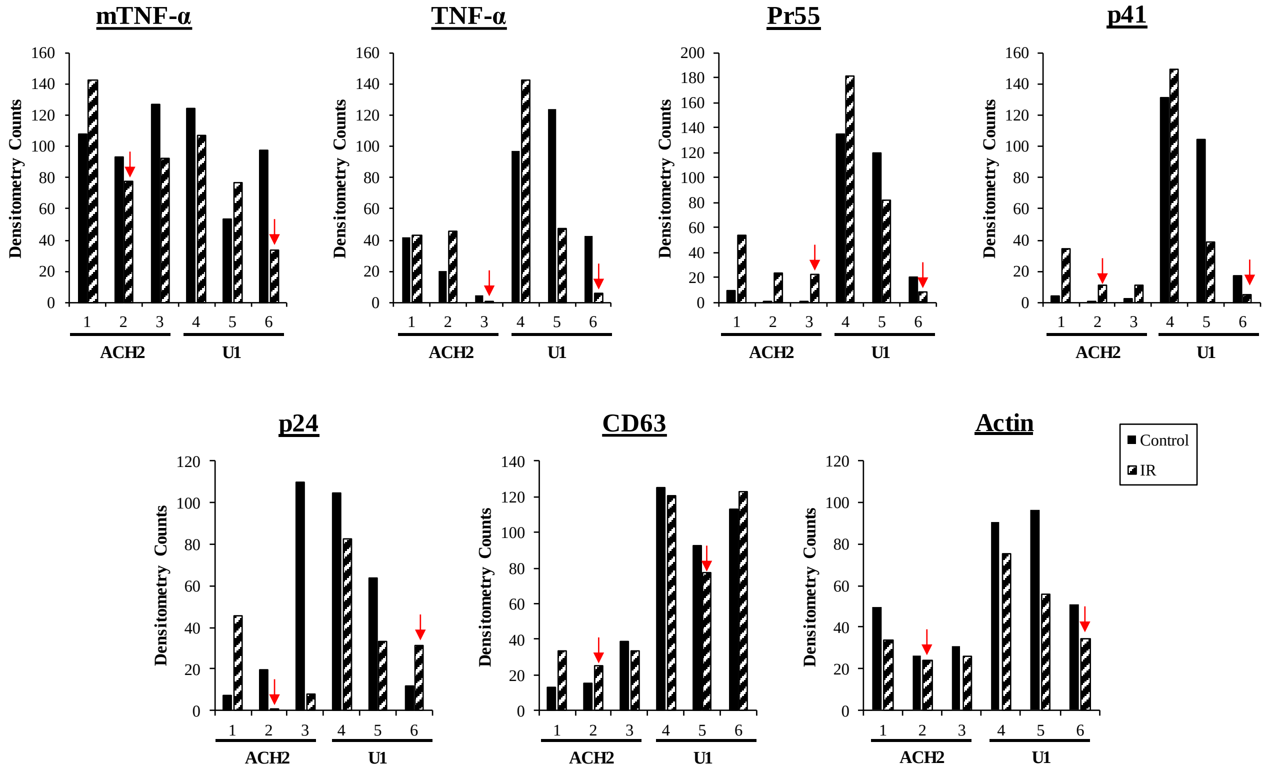

Figure S1: Quantitation of ACH2 and U1 Western blots for Figure 5A. Densitometry analysis of ACH2 and U1 cells reveal that, indeed, IR and AI treatment decreases levels of pro-inflammatory cytokines (*i.e.*, mTNF-α and TNF-α), and viral proteins (*i.e.*, Pr55, p41, and p24). Not surprisingly, the levels of EV marker CD63 and cellular protein Actin remain relatively the same with or without IR. The most significant decrease in protein levels as a response to treatment with IR in combination with AI (Rapa or INK128) was denoted by a red arrow.

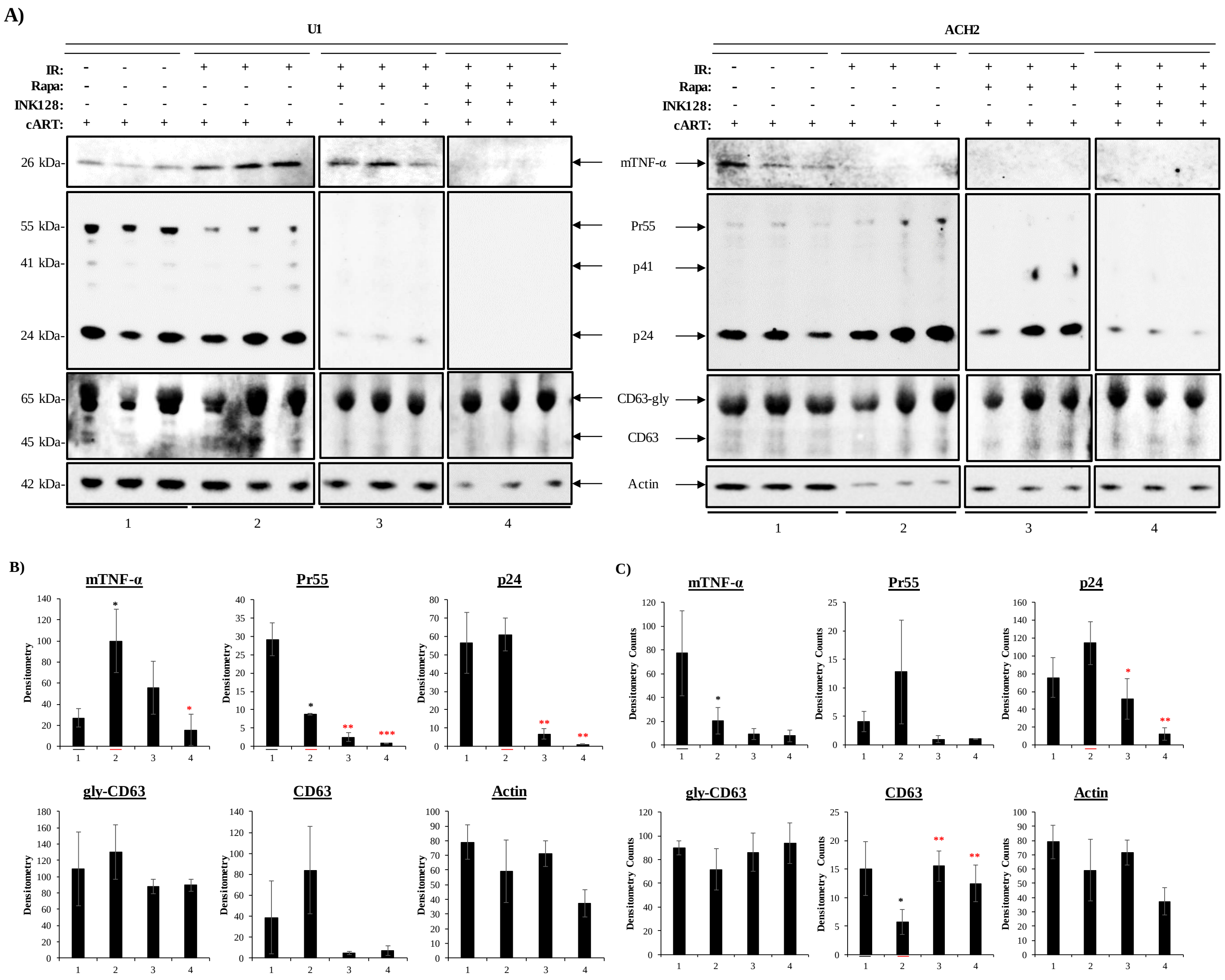

Figure S2: Validation of the effects of IR and mTORi on HIV-1 infected myeloid and T-cells. U1 (left panel) and ACH2 (right panel) were cultured and treated with IR (10 Gy), 3% FBS, and mTORi (300 nM Rapa or 300nM INK128) and allowed to incubate for 120 hours prior to isolation of EVs (using NT80/82; 30% slurry) from the supernatant material. **A**) Western blot analysis of the supernatant material normalized to 15 µLs of sample volume was performed for the detection of cytokines (*i.e.,* m-TNF-α and TNF-α), viral proteins (*i.e.,* Pr55, p41, p24), EV marker CD63, and actin. Next, densitometry analysis of each blot was performed, and biological triplicates were averaged together for **B**) U1 and **C**) ACH2 to determine standard deviation (SD; +/- 1) and statistical significance was evaluated using a two-tailed students t-test (* for p-value 0.05; ** for p-value 0.01; and *** for p-value 0.001). The black (-) or red (-) band under each lane was used to denote the statistical comparisons and correspond to the black (*) or red (*) asterisk, respectively. Densitometry analysis of gp41 bands was dropped due to the presence of low to no bands.

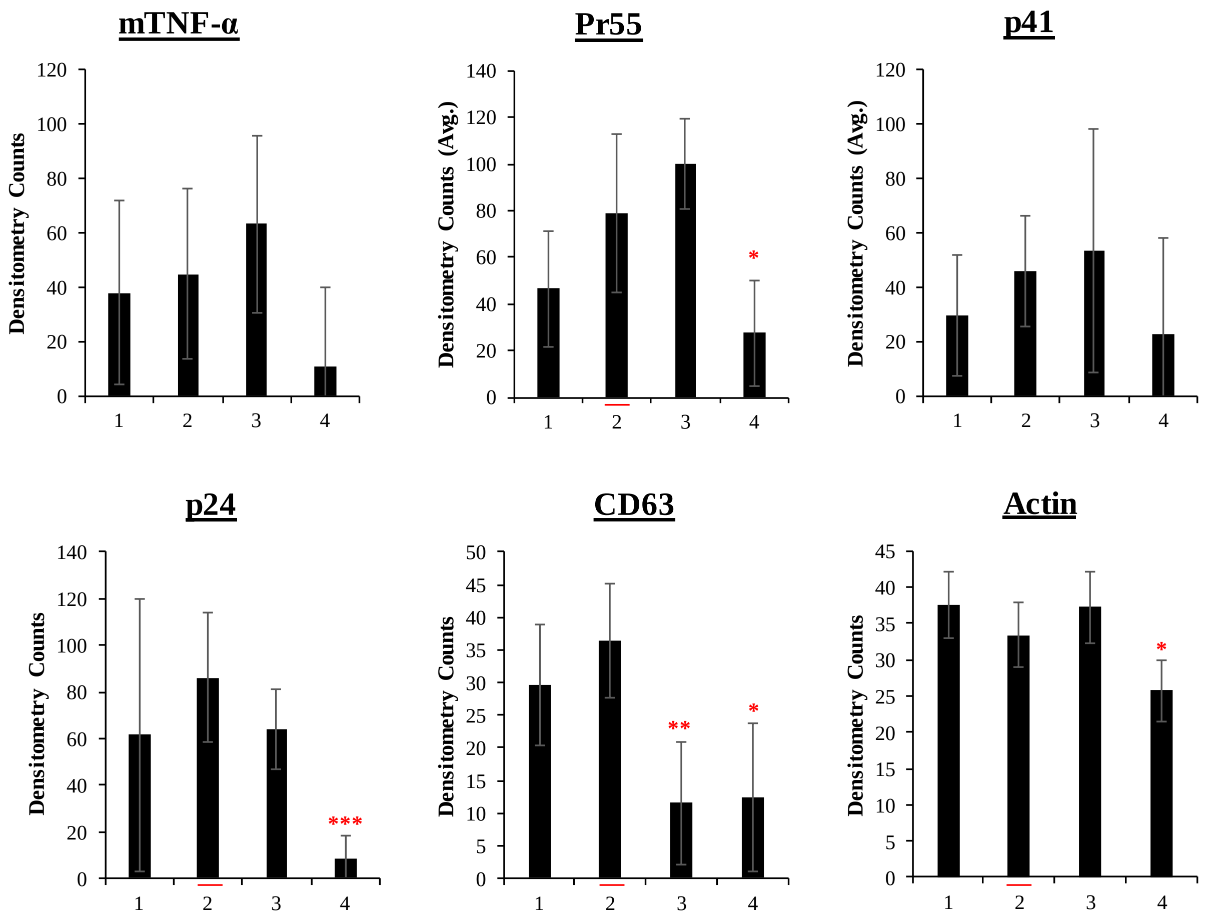

Figure S3: Quantitation of PBMC Western blots for Figure 5B. Densitometry analysis of PBMCs 1-4 was averaged together (n=4) for control (lane 1), IR (lane 2), IR/Rapa (lane 3), and IR/INK128 (lane 4) treatment conditions. Large variability exists between each PBMC, especially for PBMC 2and the lane numbers s (*i.e.*, mTNF-α and TNF-α), and viral proteins (*i.e.*, Pr55, p41, and p24). Not surprisingly, the levels of EV marker CD63 and cellular protein Actin remain relatively the same with or without IR. The greatest decrease in protein levels as a response to treatment with IR in combination with AI (Rapa or INK128) was denoted by a red arrow, and statistical significance was evaluated using a two-tailed students t-test (* for p-value 0.05; ** for p-value 0.01; and *** for p-value 0.001). The black (-) or red (-) band under each lane was used to denote the statistical comparisons and correspond to the black (*) or red (*) asterisk, respectively.

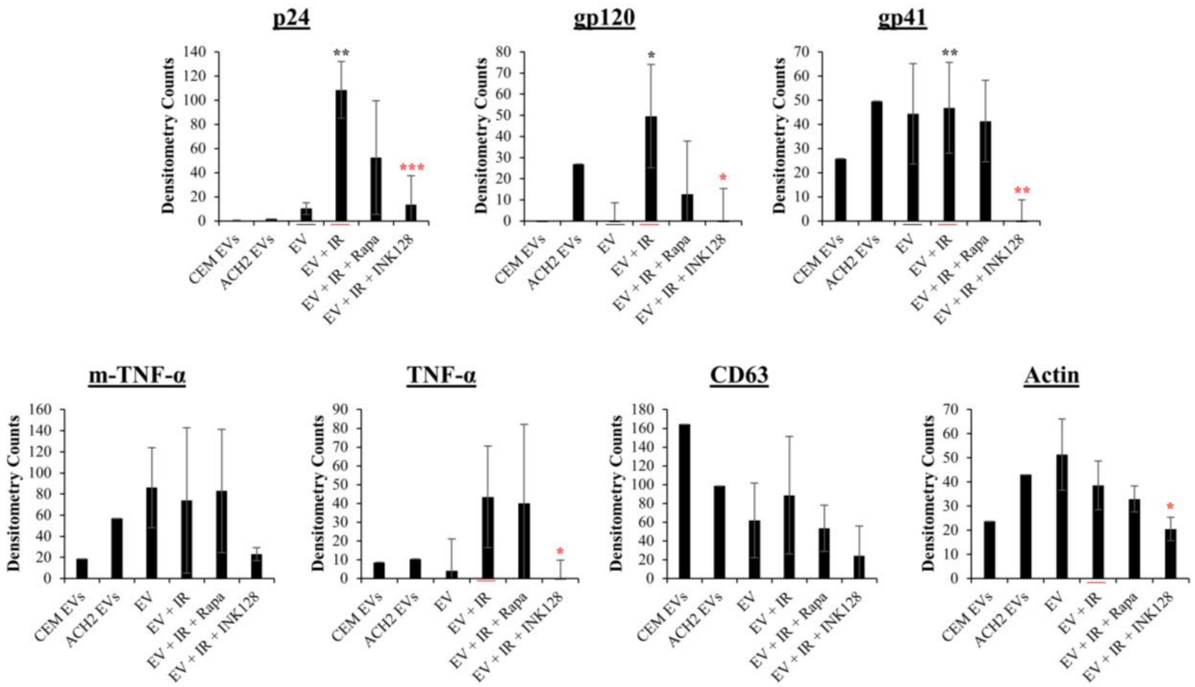

Figure S4: Densitometry Analysis of Western blots for EVs from PBMCs 5-8 on HLM-1 cells from Figure 6A. Western blots from Fig. 6A were analyzed by densitometry for each PBMCs 5-8 were averaged together (n=4) for control (lane 1), IR (lane 2), IR/Rapa (lane 3), and IR/INK128 (lane 4) treatment conditions. Statistically significant decreases in protein levels were as a response to treatment with IR/INK128, denoted by a red arrow, and statistical significance was evaluated using a two-tailed students t-test (* for p-value 0.05; ** for p-value 0.01; and *** for p-value 0.001). The black (-) or red (-) band under each lane was used to denote the statistical comparisons and correspond to the black (*) or red (*) asterisk, respectively.

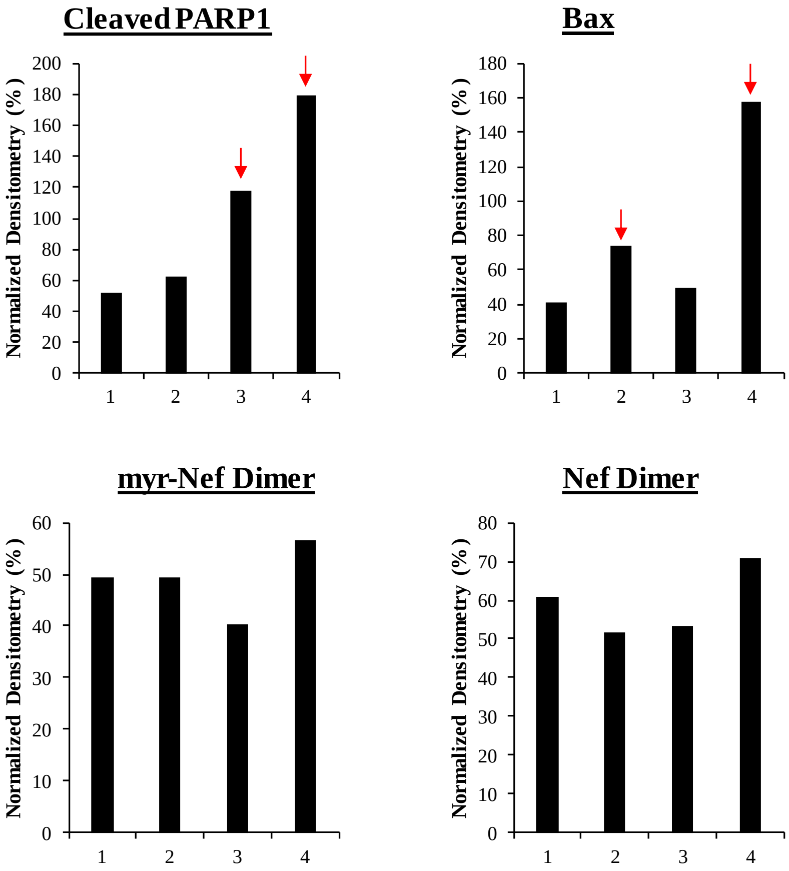

Figure S5: Densitometry Analysis of Western blots for PBMCs 9-12 in Figure 6B. Densitometry analysis (normalized to Actin) was performed of Western blots for Fig. 6B which consisted of cell pellets from PBMCs 9-12 infected with HIV-1 89.6 were treated with IR (0.5 Gy), Rapa (50 nM) or INK128 (50 nM), and 3% FBS media for 5 days prior to Western Blot analysis for markers of cell death (*i.e.*, PARP-1 and Bax) and virus (*i.e.*, Nef). Red arrows were used to indicate the largest increases in markers of cell death.
